# The metabolic origins of non-photorespiratory CO_2_ release during photosynthesis: a metabolic flux analysis

**DOI:** 10.1093/plphys/kiab076

**Published:** 2021-02-16

**Authors:** Yuan Xu, Xinyu Fu, Thomas D Sharkey, Yair Shachar-Hill, and Berkley J Walker

**Affiliations:** 1 Department of Plant Biology, Michigan State University, Michigan 48824, USA; 2 Department of Energy-Plant Research Laboratory, Michigan State University, Michigan 48824, USA; 3 Department of Biochemistry and Molecular Biology, Michigan State University, Michigan 48824, USA

## Abstract

Respiration in the light (*R***_*L*_**) releases CO_2_ in photosynthesizing leaves and is a phenomenon that occurs independently from photorespiration. Since *R***_*L*_** lowers net carbon fixation, understanding *R***_*L*_** could help improve plant carbon-use efficiency and models of crop photosynthesis. Although *R***_*L*_** was identified more than 75 years ago, its biochemical mechanisms remain unclear. To identify reactions contributing to *R***_*L*_**, we mapped metabolic fluxes in photosynthesizing source leaves of the oilseed crop and model plant camelina (*Camelina sativa*). We performed a flux analysis using isotopic labeling patterns of central metabolites during ^13^CO_2_ labeling time course, gas exchange, and carbohydrate production rate experiments. To quantify the contributions of multiple potential CO_2_ sources with statistical and biological confidence, we increased the number of metabolites measured and reduced biological and technical heterogeneity by using single mature source leaves and quickly quenching metabolism by directly injecting liquid N_2_; we then compared the goodness-of-fit between these data and data from models with alternative metabolic network structures and constraints. Our analysis predicted that *R***_*L*_** releases 5.2 μmol CO_2_ g^−1^ FW h^−1^ of CO_2_, which is relatively consistent with a value of 9.3 μmol CO_2_ g^−1^ FW h^−1^ measured by CO_2_ gas exchange. The results indicated that ≤10% of *R***_*L*_** results from TCA cycle reactions, which are widely considered to dominate *R***_*L.*_** Further analysis of the results indicated that oxidation of glucose-6-phosphate to pentose phosphate via 6-phosphogluconate (the G6P/OPP shunt) can account for >93% of CO_2_ released by *R***_*L*_**.

## Teaser

Metabolic flux analysis of photosynthesizing leaves suggests glucose-6-phosphate oxidation is the main source of non-photorespiratory CO_2_ loss.

## Introduction

During photosynthetic carbon assimilation, CO_2_ fixation is partially offset by two CO_2_ releasing processes, photorespiration, and respiration in the light (*R***_*L*_**). The biochemical origins and associated metabolism of photorespiration are well understood. However, *R***_*L*_**, defined as CO_2_ release during photosynthesis other than from photorespiration, decreases net photosynthesis by ∼10% but is much less well characterized (Tcherkez et al., 2017). The rate of *R***_*L*_** is important for understanding carbon exchange at scales ranging from a single leaf to the entire globe (Rogers et al., 2014), and as a carbon-losing process, could provide a target for increasing photosynthetic efficiency and crop yields, a grand challenge for plant biology (Grierson et al., 2011; Huber, 2011; Kromdijk and Long, 2016). Resolving the biochemical source of *R***_*L*_** is therefore vital to understanding how necessary it is to central metabolism and whether it might be engineered to increase net carbon fixation by decreasing excess carbon loss.

Traditionally, *R***_*L*_** is attributed mostly to decarboxylation reactions associated with the tricarboxylic acid (TCA) cycle and its CO_2_-releasing reactions (based on in vivo gas exchange and in vitro enzymatic and organellar transport experiments), or to a lesser degree CO_2_ release during fatty acid synthesis. However, several observations cast doubt on TCA cycle activity as an explanation for *R***_*L*_** (Atkin et al., 2000; Tcherkez et al., 2005, 2017). For example, it has long been known that only low levels of isotopic labeling are detected in TCA cycle intermediates following exposure of photosynthesizing cells to ^14^CO_2_ (Calvin and Massini, 1952), and ^14^C is not detected in CO_2_ released from leaves fed radioactively labeled sugars (Vittorio et al., 1954). More recent ^13^CO_2_ labeling experiments on shoot tissues have also shown low levels of labeling in TCA cycle intermediates (Szecowka et al., 2013; Ma et al., 2014; Abadie et al., 2017), and estimates of the rate of CO_2_ release from the TCA cycle estimated from these results are much less than *R***_*L*_** values deduced using the commonly used “Laisk” or “Kok” methods that are based on CO_2_ gas exchange from separate experimental reports (Tcherkez et al., 2017). Fatty acid synthesis may release more CO_2_ than TCA cycle reactions, but likely still much less than estimates of *R_L,_* although a formal comparison of these values has not been made (Bao et al., 2000).

**Figure 1:**
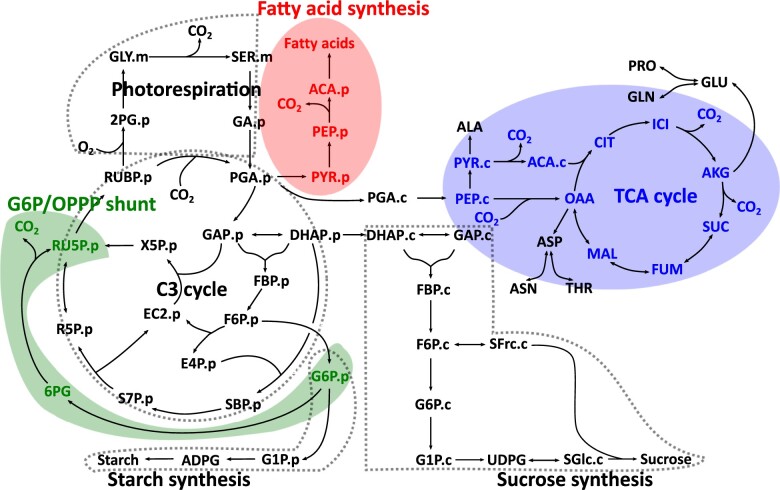
Metabolic network model of central metabolism with potential *R_L_* sources in photosynthetic *C. sativa* leaves. Metabolic network model with pathways including C_3_ cycle, photorespiration, starch synthesis, and sucrose synthesis are shown in dotted lines. Plastidic, cytosolic, and mitochondrial pools are represented by (“.p”), (“.c”), and (“.m”). Irreversible reactions are shown with single-headed arrows and reversible reactions are shown with double-headed arrows. Three possible *R_L_* sources including the TCA cycle (blue), G6P/OPPP shunt (green), and fatty acid synthesis (red) pathway are shown. All remaining abbreviations are shown in SupplementalData set S9.

The mismatch between estimated TCA cycle and fatty acid fluxes and measured *R***_*L*_** values raises the question of which alternative pathway explains this CO_2_ release. Three possible pathways for additional sources of *R***_*L*_** are shown in our flux model (Figure 1). One is the TCA-associated decarboxylation reactions. Another is the decarboxylation of pyruvate during the production of acetyl-coenzyme A (acetyl-CoA), primarily to support fatty acid biosynthesis (Williams and Randall, 1979). A third possible source is from an oxidative pentose phosphate (OPP) shunt, where glucose 6-phosphate (G6P) is converted to ribose 5-phosphate while releasing CO_2_ (G6P/OPP Shunt; Sharkey and Weise, 2016). This bypass is thought to operate to maintain the carbon balance of the C_3_ cycle, and recent simple modeling of static labeling data was consistent with the shunt contributing a large portion of the CO_2_ release measured as *R***_*L*_** (Sharkey et al., 2020). Quantitative flux approaches are especially suited for investigating the biochemical source(s) for *R***_*L*_**.

Measurements of metabolic fluxes produce a picture of functional metabolism by integrating enzyme expression, activity, and network structure. Metabolic flux analysis (MFA) is a powerful approach to measure intracellular metabolic fluxes in an in vivo biological system by combining computer models with experimental measurements (Stephanopoulos et al., 1998). MFA studies are crucial for understanding cellular and metabolic phenotypes, and have been applied to metabolic engineering (Stephanopoulos, 1999; Reed et al., 2010), biotechnology (Boghigian et al., 2010; Woo Suk and Antoniewicz, 2013), systems biology (Niklas and Heinzle, 2012), and biomedical research (Hiller and Metallo, 2013). The predominant approach for measuring metabolic fluxes using ^13^C as a labeled tracer (^13^C-MFA) has been used on microbial systems incubated with a ^13^C-labeled precursor, and requires the system to reach an isotopic and metabolic steady state. Such model-based MFA approaches were developed in the early 1990s and the techniques have been improved over the last 20 years (Vallino and Stephanopoulos, 1993; Varma and Palsson, 1994). However, this ^13^C-MFA approach cannot be applied in autotrophic plant cells to track carbon through primary fixation since ^13^CO_2_ labels all of metabolism in the steady-state, leaving no flux information in the labeling patterns of metabolites (Roscher et al., 2000; Ratcliffe and Shachar-Hill, 2006).

Dynamic labeling approaches overcome the limitations of isotopic steady state ^13^C-MFA and estimate metabolic fluxes by analyzing the time-course of label redistribution through metabolism. Isotopically nonstationary MFA (INST-MFA) estimates intracellular metabolic fluxes by modeling the dynamic isotope labeling trajectories of each carbon position in metabolic intermediates explicitly (Wiechert and Nöh, 2005). The values of metabolic fluxes in the network determined by INST-MFA are those which yield the best fit between calculated and measured labeling patterns. Label measurements are primarily made by mass spectrometry, yielding mass isotopologue distributions (MIDs). MIDs describe the proportions of molecules comprising a metabolite with zero, or progressively more, atomic carbon positions containing a heavy isotope derived from the labeled substrate. By using MIDs, INST-MFA can leave the pool size as unknowns and solve for them, avoiding inaccuracies caused by changes in absolute metabolite levels during sample quenching, extraction, and quantification (Ma et al., 2014). INST-MFA has yielded insights into physiological and regulatory aspects of cellular metabolism, with both basic biological and applied relevance (Nöh and Wiechert, 2006). Improvements in label analysis and computational modeling have made INST-MFA a powerful tool for studying autotrophic carbon metabolism in photosynthetic cells, despite the challenges presented by network complexity, compartmentation, hard-to-analyze metabolites, and biochemical knowledge gaps (Shastri and Morgan, 2005; Young, 2014). Here, we expand recent work extending INST-MFA to the analysis of vascular land plants by investigating the biochemical source of *R***_*L*_** in photosynthetic metabolism.

Past work using INST-MFA and related kinetic labeling approaches analyzed fluxes of *Arabidopsis thaliana* whole rosettes, which comprise a mix of leaves at different developmental stages (Szecowka et al., 2013; Ma et al., 2014). Leaf development affects photosynthetic capacity and fluxes through central metabolism due to the heterogeneity of leaf expansion, leaf senescence, mesophyll resistance, and the gas exchange interfaces of chloroplasts in *A. thaliana* and *Chenopodium album* (Stessman et al., 2002; Oguchi et al., 2003; Wingler et al., 2004; Bielczynski et al., 2017). Additionally, source and sink leaves have vastly different metabolic programs. To avoid the heterogeneity in photosynthetic metabolism of leaves from different developmental stages, we used single, fully expanded source leaves at similar developmental stages. We also measured labeling in additional metabolic intermediates, compared with previous studies, and non-invasively determined the sucrose export rate, providing additional experimental constraints on the carbon balance and computed flux distribution. This work also builds upon past work in individual tobacco (*Nicotiana tabacum*) leaves by measuring a more extensive set of metabolites and expanded flux modeling framework (Hasunuma et al., 2010).

Additional methodological considerations for ^13^C-MFA studies in photosynthetic tissues require minimizing the switching time between ^12^CO_2_ and ^13^CO_2_ and rapidly quenching metabolism (Badger et al., 1984; Hasunuma et al., 2010). Rapid lowering of the temperature of the entire tissue is especially important to quench metabolism. Rapid quenching is necessary since C_3_ cycle metabolic intermediates have extremely fast turnover times that are from 0.08 to 3.23 s (Arrivault et al., 2009). Both metabolic concentration and MID measurements are therefore strongly affected by quenching speed, since even a short period in the dark would affect the fluxes through these intermediates of central metabolism. Sampling C_3_ cycle intermediates during early labeling time points (from a few seconds to a few minutes) is especially important in INST-MFA, since this is when labeling patterns change rapidly and are most informative. Therefore, both switching time and quenching time must be rapid to ensure the labeling data are representative of in vivo conditions. To minimize quenching time, we sprayed liquid nitrogen directly onto the leaf in the labeling chamber to freeze it in 500 ms or less.

Understanding the source of *R***_*L*_** is critical for possibly improving the carbon-use efficiency of crop plants. The emerging oilseed crop and model plant, camelina (*Camelina sativa*) has shown considerable promise as a low input, stress tolerant oilseed crop that possesses desirable oil qualities for both nutrition and biofuel. Specifically, camelina oil contains a high level of omega-3 fatty acids, vitamin E, and antioxidants, which are important for human and animal nutrition (Petrie et al., 2014). On a dry weight basis, the oil content of camelina ranges from 30% to 40%, and camelina-oil-based blends have been tested and approved as liquid transportation fuels (Moser, 2010; Obour, 2015). Compared to other oilseed crops, camelina has a short growing season (85–100 d), allowing its use as an intercrop, and suffers fewer losses from pests (Petrie et al., 2014). In addition, useful traits can be identified and introduced into camelina because of the fully sequenced genome, well-established molecular genetic tools, and numerous resources available from its close relative, *A. thaliana* (Putnam et al., 1993; Malik et al., 2018)*.* The major limitation to widespread adoption of camelina as an oilseed crop is its modest yield, currently about half that of its relative, *Brassica napus.* This makes improving photosynthetic efficiency by reprogramming central metabolism for increased yield especially attractive (Dalal et al., 2015; Ort et al., 2015; Kromdijk and Long, 2016; Chhikara et al., 2018)

In this work, we used INST-MFA on illuminated camelina leaves to estimate the carbon fluxes of central metabolism and resolve the biochemical source of *R***_*L*_**. We found that fluxes associated with the TCA cycle are not large enough to explain the measured *R***_*L*__,_** and hypothesize that other CO_2_-releasing processes contribute to *R***_*L*_**. This hypothesis was tested by fitting metabolic ^13^C labeling kinetics to a structural model of central metabolism in which CO_2_-releasing flux was constrained by measured *R***_*L*_**. According to these simulations, we found that the G6P/OPP shunt best explained the release of CO_2_ by *R***_*L*_** and gave the best agreement between measured and simulated labeling kinetics. This work improves our understanding of the biochemical drivers for plant CO_2_ exchange and may benefit future work for improving net CO_2_ assimilation, carbon use efficiency, and crop yield in camelina and other crop plants.

## Results

### Metabolic flux mapping quantifies contributions to *R_L_*

**Figure 2:**
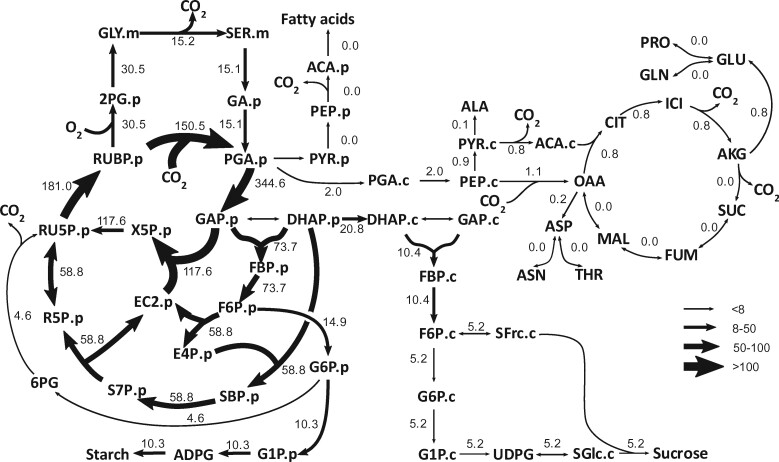
Central carbon assimilatory metabolic fluxes in photosynthetic *C. sativa* leaves without the constraint of measured *R_L_*. Fluxes are shown in numbers and also depicted by the variable width of arrows. Fluxes were estimated by ^13^C INST-MFA using the INCA software suite constrained by the metabolic network model and experimental inputs including mass isotopomer distributions of measured metabolites, net CO_2_ assimilation, starch synthesis rate, sucrose synthesis rate, and amino acid export rate. Fluxes were not constrained by measured *R_L_*. Flux units are expressed as μmol metabolite g FW^−1^ h^−1^. The model network is compartmentalized into cytosol (“.c”), which includes mitochondrial and peroxisomal reactions, plastid (“.p”), and mitochondria (“.m”). Metabolite pools (principally vacuolar) that do not become labeled on the time scale of the experiments are modeled but not shown in this figure. All remaining abbreviations are shown in Supplemental Dataset S9.

Measurements of ^13^C labeling kinetics and several input and output fluxes (introduced below) were used to estimate metabolic fluxes through central metabolism using INST-MFA. The resultant flux map (Figure 2) quantifies reaction rates through CO_2_ releasing reactions that may contribute to *R***_*L*_**. The metabolic model of central metabolism of photosynthetic camelina leaves contains the C_3_ cycle, photorespiration, starch and sucrose synthesis, as well as photorespiratory, glycolytic, and intracellular metabolic transport steps. Three potential *R***_*L*_** sources were included in the model: the TCA cycle, fatty acid synthesis, and the G6P/OPP shunt (Figure 1). Computing the best fit fluxes using the model and data was performed using the INCA software suite, which yields flux values and shows the quality of agreement between measured and predicted labeling levels for each metabolite and metabolite fragment analyzed (Young, 2014). Ninety-five percent confidence intervals of the best fit fluxes were determined using both parameter continuation and Monte Carlo methods; Supplemental Datasets S1–S8). Experimental measurements on individual leaves included the MIDs of most metabolites in the network, which were measured by a combination of three mass spectrometric approaches (see methods). Rates of carbon input and output from the central metabolic network were measured either directly (steady-state net CO_2_ assimilation and starch accumulation rate) or inferred from the labeling rate of sucrose and exudate composition to yield export rates of sucrose and amino acids (see methods). These independently determined rates accounted for steady state carbon balance (system entry = exit) and provided constraints for the flux mapping from the labeling data. Based on previous analyses of in vivo rubisco kinetics under ambient conditions, the ratio of oxygenation to carboxylation rates by rubisco (*v_o_*/*v_c_*) was constrained to be between 0.2 and 0.25 (Sharkey, 1988). The net photosynthetic rate per unit of leaf area (± sd here and throughout) was 18.3 ± 2.7 µmol m^−2^ s^−1^, and stomatal conductance to water vapor was 0.25 ± 0.06 mol m^−2^ s^−1^.

*R* **_*L*_** was estimated as the sum of non-photorespiratory fluxes estimated from the model that produced CO_2_ to be 5.2 μmol CO_2_ g^−1^ FW h^−1^, with the 95% confidence intervals of 3.4–7.5 estimated using parameter continuation (see methods for details, Supplemental Dataset S5). When the simulated data and measured labeling patterns were best fit, the major biochemical source flux for *R_L_* was the G6P/OPP shunt, which contributed 4.6 μmol CO_2_ g^−1^ FW h^−1^, i.e., 88% of *R***_*L*_** (Figure 2 and Supplemental Dataset S5). CO_2_ release from TCA-associated reactions explained the remaining 0.6 μmol CO_2_ g^−1^ FW h^−1^ of *R***_*L*_**, with phosphoenolpyruvate (PEP) carboxylation to oxaloacetate (OAA, 1.1 μmol CO_2_ g^−1^ FW h^−1^) partially offsetting cytosolic CO_2_ release from oxidative decarboxylation of pyruvate to acetyl-CoA (0.8 μmol CO_2_ g^−1^ FW h^−1^) and α-ketoglutarate decarboxylation (0.8 μmol CO_2_ g^−1^ FW h^−1^, Figure 2 and Supplemental Dataset S5). There was negligible contribution to *R***_*L*_** by CO_2_ release from oxidative decarboxylation of pyruvate, which produces acetyl-CoA for fatty acid synthesis in the plastid.

### Measurement of gas exchange, *R_L_*, and starch and sucrose synthesis rates

**Figure 3:**
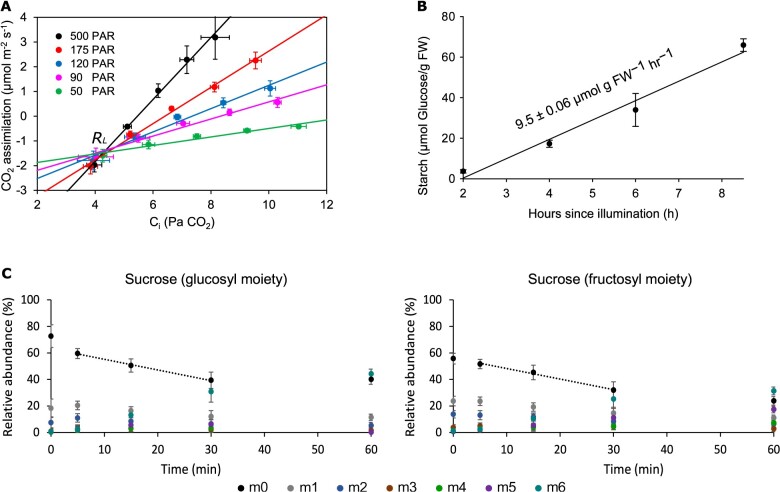
Leaf metabolism and measurement of *R_L_*, starch, and sucrose synthesis rates used to constrain flux models. A, Measurement of *R_L_*. Common intersection (Laisk) measurement of CO_2_ assimilation measured as a function of intercellular CO_2_ concentration (C_i_) used to determine *R_L_* (the Y-axis intersection point) measured at five sub‐saturating light intensities indicated by the PAR values on the plot with linear fits (A, *n* = 4, ± sd). B, Measurement of starch synthesis rate. Starch was measured from individual timepoint assays of starch content, and measurements were taken between 2 and 8.5 h after illumination during the same time of day when ^13^CO_2_ labeling experiments were performed when plants exhibited pseudo-steady state metabolism (B, *n* = 3, ± sd). C, Measurement of sucrose synthesis rate. The time course of mass isotopomer distribution of glucosyl and fructosyl moieties of sucrose determined from GC-MS peaks m/z 361 and 451 was used to determine the sucrose synthesis rate (*n* = 3, ± sd). The dotted lines represent the linear labeling of the glucosyl and fructosyl moieties of the sucrose fragments within the first 30 min.

To estimate *R***_*L*_** independently from the flux analysis, we determined *R***_*L*_** from leaf gas exchange using the common intersection or “Laisk” method (Laisk and Agu, 1977; Brooks and Farquhar, 1985; Figure 3A) to be 1.4 ± 0.2 μmol CO_2_ m^−2^ s^−1^ on a leaf area basis or 9.4 ± 1.3 μmol CO_2_ g FW^−1^ h^−1^ on a fresh-weight basis. This mean and sd translates to a 95% confidence interval between 8.1 and 10.7 μmol CO_2_ g^−1^ FW h^−1^. This range falls within the INST-MFA-estimated 95% confidence interval of *R***_*L*_** determined from the Monte-Carlo analysis (3.6–8.6 μmol CO_2_ g^−1^ FW h^−1^), but just outside the range determined using parameter continuation (3.4–7.5 μmol CO_2_ g^−1^ FW h^−1^, Supplemental Dataset S5). While the source of the difference between these two estimates of *R***_*L*_** cannot be determined exactly, it could be due to physiological differences when the measurements were made (Laisk measurements are made under low-light and low CO_2_ concentrations), assumptions made in the model structure, or could represent statistical variance inherent to the measurements themselves. Indeed, various approaches of measuring *R***_*L*_** result in slightly different absolute estimations, but estimates that are in a similar range of each other and of the Laisk and flux-predicted values outlined above (Villar et al., 1994). Given that the differences in estimates of *R_L_* between the two approaches are relatively small, relative to rates of flux through other parts of the network, and in line with differences between methods measured in past work, we are confident that these values compare reasonably well, especially given the vastly different nature of the assumptions used to arrive at their estimations.

The fluxes of net starch production, CO_2_ uptake, and sucrose export were independently determined and used as inputs to the INST-MFA, along with measurements of labeling in metabolic intermediates. The starch synthesis rate (9.5 ± 0.1 μmol glucose· g^−1^ FW h^−1^) was determined from the buildup of starch during the timeframe that labeling occurred, which was measured between 2 and 8 h after illumination (Figure 3B). The rate of sucrose synthesis (4.3 ± 1.1 μmol sucrose g^−1^ FW h^−1^) was determined from the initial, linear labeling of the glucosyl and fructosyl moieties of the sucrose fragments (Figure 3C). Thus, starch and sucrose synthesis accounted for about 72% of net carbon fixation, similar to what is found in other labeling studies (e.g., Sharkey et al., 1985).

### Fitting of metabolic fluxes to the transient labeling patterns of individual metabolites and their fragments

**Figure 4:**
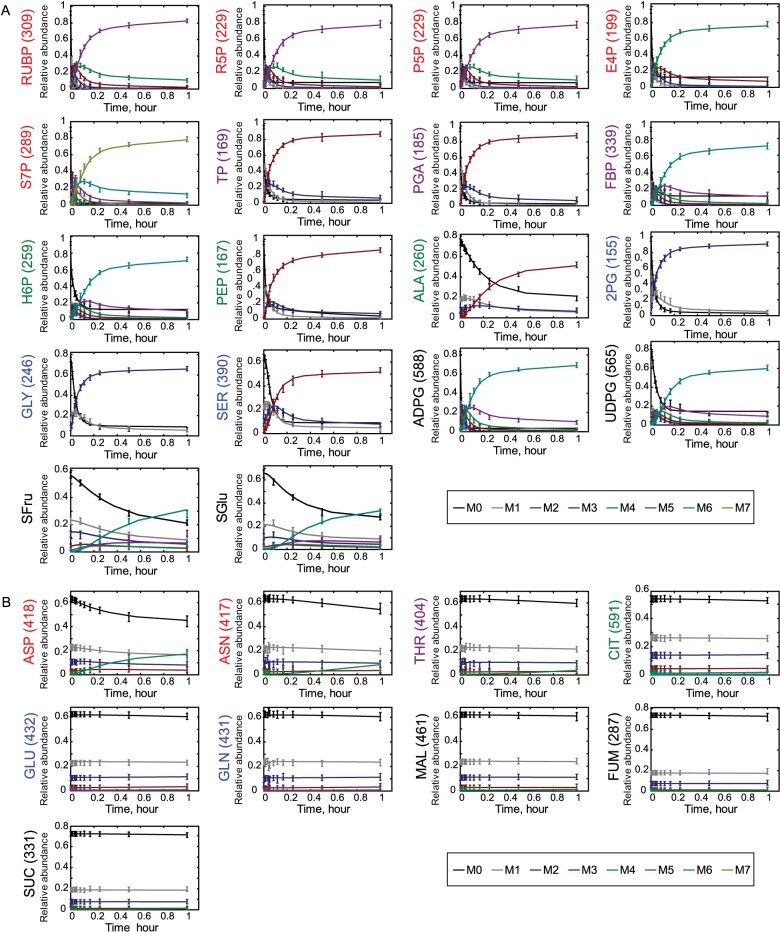
Transient ^13^CO_2_ labeling in selected metabolites. Experimentally determined isotope labeling measurements are shown as points with error bars (*n* = 3, ± sd). INST-MFA fitted mass isotopomer distributions are shown as solid lines. The nominal mass isotopomers are represented as M0 are shown in parentheses. Other labeled isotopomers are distinguished by their mass differences from M0 (M1, M2, M3, etc.). A, C_3_/Glycolysis related metabolites. Core C_3_-only intermediates (y-axis label in red); intermediates shared with glycolysis (purple); core glycolysis metabolites (G6P) and products (PEP, Ala; green); photorespiratory intermediates (blue); carbohydrate building substrates (black). B, TCA cycle related metabolites. OAA derived AA’s (y-axis label in red); Thr, which is made from Asp (purple); Citrate (green); Glu and Gln ions (blue); Malate Fumarate and Succinate (black).

The labeling of 40 separate metabolites and their molecular fragments was measured over time, and the results fitted by INST-MFA to obtain the flux map shown in Figure 2. Figure 4 shows the representative labeling data used for the flux solution resolved by individual mass isotopologues together with the kinetics simulated by the best-fit INST-MFA flux values presented in Figure 2. Only representative molecular ions are shown here; the labeling data for all measured ions is shown in Supplemental Figure S1. Overall, the fitted flux solution is in good agreement with the measured labeling kinetics. The goodness of fit is quantified as the sum of weighted squared residuals (SSR), which quantifies the total divergence between measured and simulated kinetics, and is minimized to obtain the flux solution (Young, 2014). The SSR of this flux solution and other scenarios are discussed in more detail below (Table 1).

**Table 1:** SSR and *v*_o_/*v*_c_ for models of not forced, forced CO_2_ non-photorespiratory CO_2_ release, forced TCA, and forced fatty acid with and without constraints of *v_o_/v_c_*.

	Unconstrained *v_o_/v_c_*				Constrained *v_o_/v_c_* > 0.2			
	Not Forced	Forced CO_2_.np	Forced TCA	Forced Fatty Acid	Not Forced	Forced CO_2_.np	Forced TCA	Forced Fatty Acid
**Total SSR**	803	804	1202	891	820	834	1261	903
***v_o_***	12	17	16	23	31	31	31	31
***v_c_***	135	133	147	150	150	155	155	154
***v_o_/v_c_***	0.09	0.13	0.11	0.15	0.20	0.20	0.20	0.20

Global best fit SSR were calculated by parameter continuation analysis. Four possible scenarios were tested by forcing the non-photorespiratory CO_2_ to equal the measured *R_L_*: (1) Unconstrained *R_L_*; (2) a mix of the TCA cycle, fatty acid synthesis and the G6P/OPP shunt explained *R_L_* (Forced CO_2_.np); (3) The TCA cycle alone explained *R_L_* (Forced TCA); (4) Fatty acid synthesis alone explained *R_L_* (Forced fatty acid), with and without the constraint *v*_o_/*v*_c_. *v*_c_, biochemical velocity of rates of rubisco carboxylation; *v*_o_, biochemical velocity of rates of rubisco oxygenation; constraint *v*_o_/*v*_c_ was set in the range of 0.2–0.25. Individual SRES for each metabolite contributed to SSR are shown in Supplemental Figure S3.

These plots qualitatively reflect the flux estimates from Figure 2. Specifically, TCA-cycle intermediates showed slow labeling, with less than 5% total label enrichment after 1 h (Figure 4, Supplemental Figures S1 and S2), in comparison to intermediates of the C_3_ cycle, photorespiration, and starch and sucrose synthesis pathways, which showed substantial labeling. Slow labeling of TCA cycle intermediates is consistent with previous studies in Arabidopsis (Arrivault et al., 2009; Ma et al., 2014).

### C_3_ cycle and photorespiratory intermediates

The best-fit values of fluxes of the C_3_ cycle and photorespiration are most strongly influenced by the measured CO_2_ assimilation rate and the MIDs of the metabolic intermediates involved. All C_3_ cycle intermediates showed fast labeling at the earliest time point, starting at 30 s with a rapid decrease in the unlabeled M0 isotopologue fraction (Figure 4A, Supplemental Figures S1A and S2). The rapid labeling patterns are consistent with the short half-lives of C_3_ cycle intermediates (Arrivault et al., 2009; Hasunuma et al., 2010; Szecowka et al., 2013; Ma et al., 2014). Throughout the labeling period, there were similar patterns of a monotonic decrease in the unlabeled isotopologue fraction, a rise in the fully labeled isotopologue fraction, and an increase followed by a fall in the partially labeled isotopologue fractions. Fructose 1,6-bisphosphate (FBP) and glucose 6-phosphate (G6P)/fructose 6-phosphate (F6P) labeled more slowly than other C_3_ cycle intermediates. This is to be expected since FBP and G6P/F6P are intermediates in both sucrose and starch synthesis pathways, and the sucrose synthesis pathway occurs outside of the chloroplast, meaning that turnovers of these compounds are not determined by the higher-flux of the C_3_ cycle alone.

Most C_3_ cycle intermediates were heavily, but not fully, labeled in 60 min, with final enrichments reaching between 85% and 93% (Supplemental Figure S2). Sedoeheptulose-7-phosphate (S7P) was the most highly labeled metabolite, with ^13^C enrichment of 93% after 60 min. Ribulose 1,5-bisphosphate (RuBP), 3-phosphoglycerate (3PGA), glyceraldehyde 3-phosphate (GAP)/dihydroxyacetone phosphate (DHAP), erythrose 4-phosphate (E4P), ribulose 5-phosphate (Ru5P)/xyulose 5-phosphate (Xu5P), and ribose 5-phosphate (R5P) all have ^13^C enrichments of more than 90% (Supplemental Figure S2). G6P and F6P reached 85% and 86% labeling, respectively. The labeling of adenosine diphosphate (ADP)-glucose (ADPG, 92%) and uridine diphosphate glucose (UDPG, 78%), likely reflect a mixture of stromal and cytosolic G6P and F6P (Supplemental Figure S2). For measured photorespiratory intermediates, 2-phosphoglycolate (2PG), glycine, and serine showed rapid labeling and high ^13^C enrichments after one hour and which are comparable to C_3_ cycle intermediates (2PG: 92%; Gly: 71%; Ser: 68%, Supplemental Figure S2).

### Using further INST-MFA simulations to test hypotheses concerning the sources of *R_L_*

While the best-fit flux map assigned the majority of *R***_*L*_** to the oxidative steps of the OPP/G6P shunt, we tested the robustness of this finding by assessing the extent to which alternative hypotheses for the source of *R***_*L*_** affect the goodness of fit between the flux solution and measured data. To do this, the model was successively constrained to account for the independently measured *R***_*L*_** by flux through each of the putative CO_2_ releasing reactions of Figure 1. The resulting change to the total goodness of fit, represented by the SSR (low SSR’s representing better fits and high SSR’s representing worse fits), was then quantified by how much the total goodness of fit suffered based on each hypothesis. The initial simulations revealed that the flux solution of the ratio of rubisco oxygenation reaction relative to carboxylation (*v_o_*/*v_c_*) ranged between 0.09 and 0.15 (Table 1, Supplemental Figure S3, and Supplemental Datasets S1-S4). Since this ratio is lower than what is generally accepted as the ratio in vivo, we performed a second set of scenarios, additionally constraining *v_o_*/*v_c_* to the range 0.2–0.25, which is in line with previous estimates (Table 1, Supplemental Figure S3, and Supplemental Datasets S5–S8; Sharkey, 1988; Evans, 1999; Terashima et al., 2011).

We will now discuss the results from each scenario. To help differentiate the assumptions made for each flux solution, we will use “forced” when we set values related to CO_2_ contributing to *R_L_* and “constrained” when we have set the ratio of *v_o_*/*v_c_* to a particular range (0.2–0.25).

**Figure 5:**
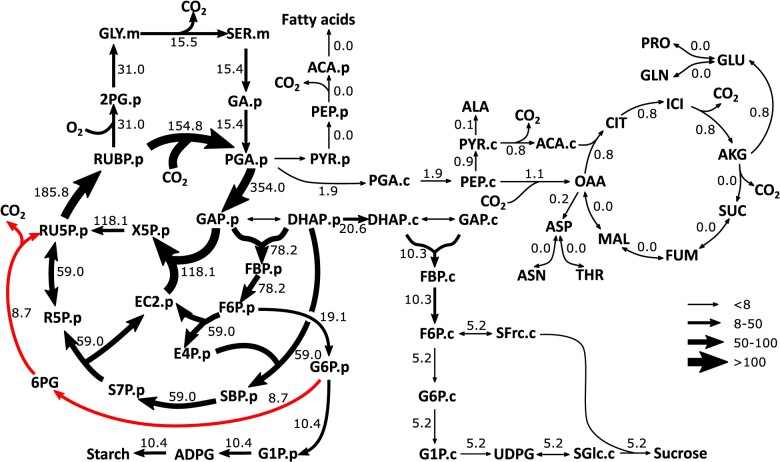
Central carbon assimilatory metabolic fluxes in photosynthetic *C. sativa* leaves constrained by the total non-photorespiratory CO_2_ release (CO_2_.np) to be equal to the measured *R_L_*. Fluxes are shown in numbers and corresponding variable width of arrows. Fluxes were estimated by ^13^C INST-MFA by INCA using the metabolic network model and experimental inputs including mass isotopomer distributions of measured metabolites, net CO_2_ assimilation, starch synthesis rate, sucrose synthesis rate, and was constrained by measured *R_L_* and *v_o_/v_c_*. Flux unit was expressed by μmol metabolite·gFW^−1^·h^−1^. The model was compartmentalized into cytosol (“.c”), plastid (“.p”), and mitochondria (“.m”). The red arrow represents that the major biochemical source flux for *R_L_* was the G6P/OPP shunt, which contributed 94% of *R***_*L*_**. All remaining abbreviations are shown in Supplemental Dataset S9.

**Figure 6:**
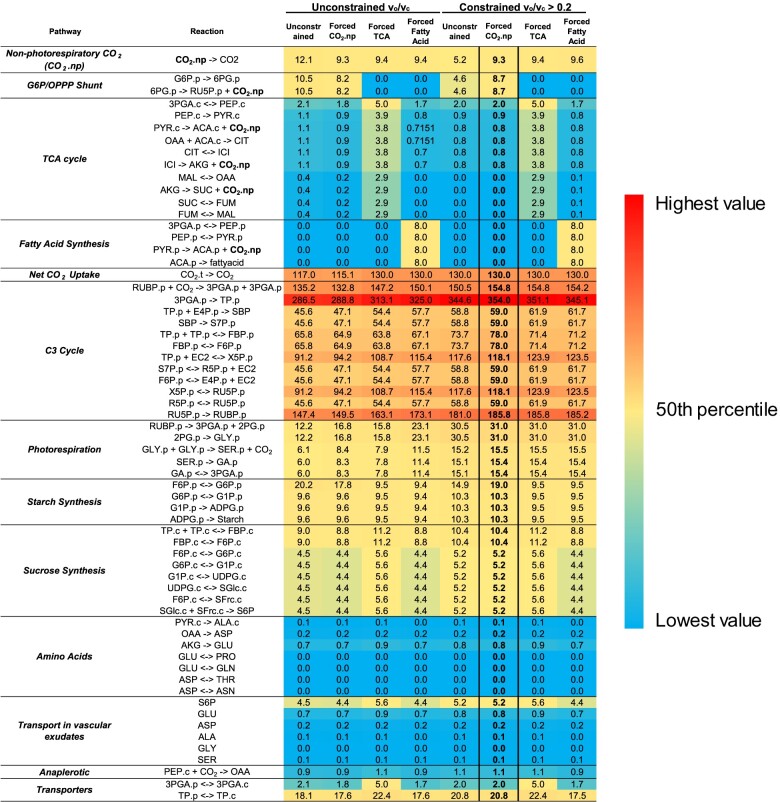
Heatmap for net fluxes for models of normal, forced CO_2_ non-photorespiratory CO_2_ release, forced G6P/OPPP shunt, forced TCA, and forced fatty acid with and without constraints of *v_o_/v_c_*. Net fluxes were determined by ^13^C INST-MFA by INCA using the metabolic network model and experimental inputs including mass isotopomer distributions of measured metabolites, net CO_2_ assimilation, starch synthesis rate, and sucrose synthesis rate. Flux unit was expressed by μmol metabolite·gFW^−1^·h^−1^. Four possible scenarios, with and without the constraint of *v_o_/v_c_*_,_ were tested by forcing the non-photorespiratory CO_2_ to equal the measured *R_L_*: (1) a mix of the TCA cycle, fatty acid synthesis and the G6P/OPPP shunt explained *R_L_* (Forced CO_2_.np); (2) G6P/OPPP shunt alone caused *R_L_* (Forced TCA); (3) The TCA cycle alone caused *R_L_* (Forced TCA); (4) Fatty acid synthesis alone caused *R_L_* (Forced fatty acid). *v_c_*, biochemical velocity of rates of rubisco carboxylation; *v_o_*, biochemical velocity of rates of rubisco oxygenation; constraint *v_o_/v_c_* was in the range of 0.2 to 0.25. The fluxes for the model of forced CO_2_.np with constraint *v_o_/v_c_* as shown in Figure 5 were shown in bold. Sucrose and amino acid transport fluxes in vascular exudates were calculated by measured sucrose and amino acid levels in vascular exudates with the estimated flux of S6P + 0.1618*GLU + 0.0425*ASP + 0.01137*ALA.c + 0.003443*dummyGLY + 0.01997*dummySER -> Sink. All abbreviations are shown in Supplemental Dataset S9.

When we forced the total non-photorespiratory CO_2_ release (CO_2_.np) to be equal to the measured *R****_L_***(Supplemental Dataset S6), the G6P/OPP shunt pathway (8.7 μmol CO_2_ g FW^−1^ h^−1^) was the primary pathway explaining non-photorespiratory release (9.3 μmol CO_2_ g FW^−1^ h^−1^), contributing 94% of *R****_L,_***whereas TCA (0.6 μmol CO_2_ g FW^−1^ h^−1^) and fatty acid pathways (0.0 μmol CO_2_ g FW^−1^ h^−1^) contributed 6% and 0%, respectively (Figures 5 and 6, Supplemental Dataset S6). This result further supports that the primary *R***_*L*_** source, determined by INST-MFA, is from the G6P/OPP shunt and not TCA-cycle or fatty acid synthesis related fluxes. The global best fit SSR and the local fit for individual metabolites had similar or slightly increased squared residuals (SRES) compared to unconstrained fit (Table 1, Supplemental Figure S3).

When CO_2_ release from the TCA cycle was forced to match the measured *R***_*L*_** (to test the hypothesis that all the *R***_*L*_** came from the TCA cycle), the global best fit was negatively impacted with the SSR increased by >53% from 820 to 1261 (Table 1). In the local fits, citrate was the metabolite with the biggest increase of SRES, from 0.6 to 26.9, with a more than 44-fold increase over the unconstrained model (Supplemental Figure S3), indicating the fitting for citrate is much worse when TCA flux is set equal to *R***_*L*_**. The labeling kinetics of citrate did not support hypothetical CO_2_ loss from *R***_*L*_**. In addition, the labeling patterns for all TCA cycle intermediates were slow and had low labeling proportions after 1 h (Figure 4B, Supplemental Figures S2B and S1B) that were consistent with previous studies (Tcherkez et al., 2005; Tcherkez et al., 2009; Ma et al., 2014). The total CO_2_ release from TCA in *Arabidopsis* was reported to be only 0.4 μmol CO_2_ g^−1^ FW h^−1^ (<1% to carboxylation flux (Ma et al., 2014)), which is low relative to our measured *R***_*L*_** value. These results strengthen the case that the labeling kinetics of TCA does not support the magnitude of CO_2_ loss from *R***_*L*_**. Furthermore, the regions of fit specific to the C_3_ cycle were impacted, as SRES of C_3_ cycle intermediates all increased in the forced TCA model (Table 1, Supplemental Figure S3). Because C_3_ cycle intermediates are closest to the labeling entry point, their fits were expected to be better compared to downstream metabolites, which could cause labeling to be impacted by other aspects of central metabolism not considered in the model. Therefore, the worse fits of C_3_ cycle indicate that the TCA cycle is not the primary pathway responsible for *R***_*L*_**. Based on these results, the possibility that *R***_*L*_** came exclusively from TCA-related activity is not supported by the experimental data.

By forcing the flux through fatty acid synthesis to match the measured *R***_*L*_**, we then tested the hypothesis that *R***_*L*_** is explained by CO_2_ release from pyruvate decarboxylation to acetyl-CoA. The global fit of fatty acid-forcing scenario is worse than the unconstrained fit. The SSR for fatty acid-forcing scenario is 903, which is larger than the SSR for unconstrained model of 820 (Table 1). Most metabolites have slightly increased SRES compared to the unconstrained model. The fits specific to intermediates of the C_3_ cycle, photorespiration, and sucrose synthesis are affected by forcing fatty acid synthesis to account for *R_L_* (Supplemental Figure S3). These changes in model fit are higher than the fit produced when flux was partitioned through the G6P/OPP shunt, but these fits further give us an estimate of the amount of fatty acid flux needed to explain *R***_*L*_**, which can be compared to previous estimates of fatty acid synthesis and turnover.

## Discussion

The results from this flux mapping approach point to the oxidative decarboxylation of 6-phosphogluconate in the OPP shunt as the dominant (93%) source of non-photorespiratory CO_2_ release in photosynthesizing source leaves, with minor contributions from reactions associated with the TCA cycle and even less from fatty acid synthesis. Interestingly, this approach estimates G6P/OPP activity as being ∼6% that of rubisco activity, which is close to the 5% activity suggested from independent estimates of G6P/OPP activity in unstressed poplar (Sharkey and Weise, 2016; Sharkey et al., 2020).

Our findings do not contradict the observations that TCA cycle enzyme activity is important to maintain net photosynthetic rates—only that the absolute fluxes through these pathways are not large enough to explain *R_L_*. For example, the findings and discussions of past work indicate that mitochondrial TCA cycle enzyme activity is necessary for optimal photosynthetic rates (Nunes-Nesi et al., 2005; Nunes-Nesi et al., 2008, 2011). These observations highlight the importance of enzymes like mitochondrial malate dehydrogenase and its involvement in the malate valve. The malate valve is vital for balancing ATP and reductant (NADPH and Ferredoxin) supply from the light reactions with metabolic demand from central metabolism, but a quantitative analysis reveals total flux needed for this energy balancing is only a small fraction of total energy flux (Walker et al., 2020). Since carbon and energy fluxes are roughly proportional to each other in illuminated leaf metabolism, this example highlights that fluxes can be important, but still not comprise a large portion of an overall flux (Noctor and Foyer, 1998).

These methods and our model for INST-MFA analysis of camelina leaves allow us to identify the source of *R***_*L*_** and potential targets for engineering increased productivity through future transgenic manipulation. Both the carbon use efficiency and the nutrient use capacity can be increased to improve crop yield and production and to maintain an appropriate C:N balance (Lawlor, 2002). *R***_*L*_** is potentially important for the relationship between basic metabolic processes and plant biomass production because nutrients are assimilated in leaves using carbon backbones that are contributed by respiratory metabolism (Tcherkez et al., 2017). Identifying *R***_*L*_** metabolism that can be manipulated and optimized can therefore potentially improve camelina net CO_2_ assimilation and crop yield. Given the large potential flux and carbon loss through the G6P/OPP shunt, this loss could potentially be minimized by manipulating the first step in the pathway. Among all possible sources of *R***_*L*_** CO_2_ release, a reasonable target to improve net CO_2_ assimilation would be the G6P/OPP shunt.

Given the large potential flux and carbon loss through the G6P/OPP shunt, what would its metabolic purpose be, and could this loss be minimized? The purpose of this apparently futile cycle is not clear, but the G6P/OPP shunt may operate to continuously and flexibly replenish C_3_ cycle intermediates, especially during the induction of photosynthesis or during transitions in photosynthetic or photorespiratory rates (Sharkey and Weise, 2016; Preiser, 2020; Sharkey et al., 2020). Besides replenishing C_3_ intermediates, the OPP shunt would also affect C_5_ intermediates that can be regenerated to C_6_ and C_3_ glycolytic intermediates, and energy balancing. These questions could be resolved in future work investigating the effects of genetically perturbing the G6P/OPP shunt and investigating the consequences. We now examine the remaining findings of interest revealed in our flux analysis in more detail, both related to, and tangential to, elucidating the source of *R_L_*.

### Fatty acid synthesis cannot explain *R_L_*

According to our INST-MFA, fatty acid turnover would need to proceed at a rate of 8 μmol CO_2_ gFW^−1^ h^−1^ to explain *R***_*L*_** (Figure 6, Supplemental Dataset S8), but is there support for this high rate of fatty acid turnover? Fatty acid turnover occurs constitutively throughout leaf growth and development, and the rate of fatty acid breakdown in leaves is estimated to be 1.6%–4% per day (Bao et al., 2000; Bonaventure et al., 2004; Yang and Ohlrogge, 2009; Troncoso-Ponce et al., 2013). In fully expanded leaves, the breakdown of fatty acids is balanced by de novo fatty acid synthesis. For every molecule of palmitic acid (16:0) produced by the fatty acid synthase reaction, eight molecules of CO_2_ are released during the oxidative decarboxylation of pyruvate to acetyl-CoA in the plastids (Williams and Randall, 1979). Assuming the highest fatty acid content measured in Arabidopsis leaves during development (43 μg fatty acids cm^−2^ leaf area, Yang and Ohlrogge, 2009), we estimated that fully expanded leaves would need to make 0.2–0.4 μg fatty acids cm^−2^ leaf area s^−1^ in a 12-h d to maintain the 1.6%–4% turnover rate. The CO_2_ release from these rates of fatty acid synthesis is 0.005–0.012 μmol CO_2_ m^−2^ s^−1^, which is equivalent to 0.09–0.23 μmol g FW^−1^ h^−1^. These rates of measured turnover are one to two orders of magnitude lower than those required to explain measured rates of *R***_*L*_**, indicating that fatty acid synthesis is unlikely to explain *R***_*L*_**.

### Starch and sucrose synthesis and compartmentalization

Starch and sucrose synthesis rates are the main output fluxes of carbon metabolism in most plants, and so are required to constrain the modeling. Starch and sucrose synthesis rates were linear during the time periods where labeling was performed, indicating that experiments were in a metabolic steady-state. Starch content was measured from 2 to 8.5 h after illumination, during which period the labeling experiments were conducted (Figure 3B). The sucrose synthesis rate was calculated from the ^13^C labeling kinetics of glucosyl (SGlc) and fructosyl (SFrc) moieties of sucrose and the level of sucrose in the leaf (Figure 3C, Supplemental Figure S4). The measured rate of starch synthesis (9.5 ± 0.06 μmol glucose g^−1^ FW^−1^ h^−1^) in camelina was about two times the rate of sucrose synthesis (4.3 ± 1.08 μmol sucrose g^−1^ FW^−1^ h^−1^). This result is similar to the *Phaseolus vulgaris* study indicating that the starch synthesis rate was between one and two times the sucrose synthesis rate (Sharkey et al., 1985).

The labeling pattern of ADP-glucose (ADPG) and UDP-glucose (UDPG) can help resolve metabolite compartmentalization since ADPG is the precursor for starch biosynthesis in the chloroplast and UDPG is the precursor of sucrose biosynthesis in the cytosol. Starch is synthesized in the chloroplast from ADPG, F6P, G6P, and glucose-1-phosphate (G1P), while sucrose is synthesized in the cytosol from UDGP, FBP, F6P, G6P, and G1P. Because the other intermediates of starch and sucrose synthesis are common to both processes, ADPG and UDPG are the most characteristic intermediates that reveal metabolic subcellular compartmentation. ADPG showed fast labeling and small inactive pool comparable to C_3_ cycle intermediates, with more than 94% ^13^C enrichment in 60 min (Supplemental Figures S2 and S5). However, UDPG showed slower labeling and a substantial inactive pool so that labeling reached only 80% ^13^C enrichment in 60 min (Supplemental Figures S2 and S5). These results are consistent with the different subcellular compartmentation of starch and sucrose synthesis, and with the measured starch and sucrose synthesis rates.

Both the glucosyl and fructosyl moieties of sucrose were gradually labeled over the first 30 min, but the speed of labeling then slowed after 30 min (Figure 4A). The unlabeled M0 isotopologue of glucosyl and fructosyl moieties of sucrose decreased linearly to about 40% within the first 30 min, and then reached a plateau after 30 min. This labeling pattern suggests that there are two pools of sucrose in the leaf, 60% within an active pool involved in sucrose synthesis and export, and 40% within a pool that does not turn over substantially on the time scale of the labeling experiments. This likely represents a vacuolar storage pool that may not be involved in daytime metabolism, or which exports vascular exudate. Vacuolar storage explaining the active sucrose pool size is in line with past work using non-aqueous fractionation, where 50% of leaf sucrose was found in the vacuole (Szecowka et al., 2013).

### Organic acids, amino acids, and the reversibility of the TCA cycle

While intermediates of the C_3_ cycle, photorespiration, and starch and sucrose biosynthesis pathways showed substantial ^13^C labeling, the TCA cycle intermediates analyzed, and most amino acids derived from them, showed very little labeling within 60 min. The carbon skeletons of most of the abundant organic and amino acids are derived from 3PGA, PEP and TCA cycle intermediates in the cytosol (Figure 1). PEP showed fast labeling and high ^13^C enrichment within one hour (90%, Supplemental Figure S2), but most organic and amino acids showed much slower labeling patterns and low ^13^C enrichment after one hour (Figure 4B, Supplemental Figures S2 and S1B). All measured TCA cycle intermediates including citrate, succinate, fumarate, and malate showed less than 5% labeling in 1 h, which is consistent with previous studies in Arabidopsis (Arrivault et al., 2009; Ma et al., 2014). This may be caused by the presence of large vacuolar pools of organic acids that turn over very slowly, as well as low fluxes during photosynthesis through the active (cytosolic and mitochondrial) pools of the same organic acids, which are TCA intermediates. To provide an estimate of TCA cycle output fluxes, and thus estimate the internal TCA fluxes, the ratio of sucrose to amino acids in vascular exudate was measured and used to constrain flux through the noncyclic TCA pathway (Supplemental Table S1). The labeling speed and ^13^C enrichment of amino acids varied (Figure 4B, Supplemental Figures S2 and S1B). Most amino acids showed very slow labeling with <5% of ^13^C enrichment in one hour. However, alanine and aspartate, which are synthesized by the transamination of pyruvate and oxaloacetate, respectively, showed faster labeling and high ^13^C enrichment in one hour (alanine (Ala): 71%; aspartate (Asp): 33%; Figure 4B, Supplemental Figures S2 and S1B). Glutamate (Glu) and its downstream amino acids glutamine (Gln) and proline (Pro), which are made from the TCA cycle intermediate α-ketoglutarate, showed slower labeling and lower ^13^C enrichment at one hour (Figure 4B, Supplemental Figure S1B).

The labeling kinetics of the TCA cycle intermediates (and derivatives like amino acids) provides key insights into the reversibility of individual TCA cycle reactions under steady-state photosynthetic rates. For example, The OAA-derived amino acids acquired label slowly, and almost exclusively in the form of fully ^13^C-labeled isotopologues, indicating that OAA was fully labeled (Figure 4B, Supplemental Figure S1B). Labeling appeared first in Asp and then in its product Asn, and more slowly in Thr, which is made from Asp at a slower rate than Asn. These different labeling rates likely occur because metabolic demand for Thr is lower than for Asn, which is both more abundant than Thr in proteins and is used as a substrate for amino-transferase reactions (Figure 4B, Supplemental Figures S2 and S1B). Asp, Asn and Thr are the eventual products of PEP carboxylase using fully labeled OAA and CO_2_: ^13^CO_2_ + [^13^C_3_]PEP è [^13^C_4_]OAA. Complete labeling of OAA is consistent with the observation that PEP becomes rapidly and almost completely labeled after 1 h (Figure 4A, Supplemental Figure S1A). Since OAA becomes labeled, the absence of noticeable labeling in citrate means that citrate synthase flux is very small in comparison to the [citrate]. Products downstream of citrate, like Glu and Gln ions (labeled Glx), are not substantially labeled despite the fact that the export of Glx in the phloem requires net synthesis of Glx and therefore a net flux from citrate to AKG.

In conclusion, we applied ^13^C-labeling techniques interpreted with INST-MFA to quantitatively describe central metabolic fluxes in *C. sativa* in planta. We developed an improved ^13^C INST-MFA system for fast quenching during labeling. Based on these results, the G6P/OPP shunt pathway—and not TCA cycle-related fluxes—explains the majority of *R***_*L*_**.

## Materials and methods

### Chemical reagents

Authentic standards and ^13^CO_2_ (99.0 atom % ^13^C) were purchased from Sigma-Aldrich, Cayman Chemical, and Omicron Biochemicals Inc. HPLC-grade chloroform and methanol were purchased from Sigma-Aldrich.

### Plant growth

Wild-type *C. sativa* ecotype Suneson was grown under 8/16-h d/night cycles, under a light intensity of 500 μmol m^−2^ s^−1^, temperature of 22°C, and 50% relative humidity (RH). Fully expanded source leaves from 4-week-old pre-bolting plants were used for gas exchange measurements, ^13^CO_2_ labeling experiments, and quantification of leaf sucrose and starch and vascular sucrose and amino acids.

### *R_L_* measurement

The photorespiratory CO_2_ compensation point and *R***_*L*_** were measured using the common intersection (or Laisk) method (Laisk and Agu, 1977; Walker et al., 2016). The youngest fully expanded leaves of 4-week-old plants were used for gas exchange measurement by a LI-COR 6800 (LI-COR Biosciences, Lincoln, NE, USA). The reference CO_2_ was set to 400 µL L^−1^ (v/v), chamber temperature was set to 22°C, and RH was 70%. Four biological replicates of common intersection measurements were made at 50, 90, 120, 175, and 500 photosynthetically active radiation (PAR) from the linear fits of CO_2_ response curves measured between 30 and 400 µL L^−1^ (v/v) [CO_2_]. *R***_*L*_** was estimated using the Excel‐based tool described in Walker et al. (2016).

### Gas exchange and ^13^CO_2_ labeling with a fast atmospheric switching and quenching system

To minimize changes in metabolism during labeling and quenching experiments, a fast quenching (0.1–0.5 s) ^13^CO_2_ labeling system was developed (Supplemental Figure S6). This system consisted of a LI-COR 6800 (LI‐COR Biosciences, Lincoln, NE, USA) with a modified 9 cm^2^ cuvette, which included a hole on the lower side of the chamber to allow for insertion of a liquid nitrogen cryospray nozzle (Wallach UltraFreeze, Cooper Surgical, Trumbull CT). This hole was sealed with a stopper of adhesive putty during the initial gas exchange and labeling. Preliminary measurements indicated that the positive pressure of the cuvette was high enough that leaks during this time emanated from the chamber and the internal gas concentrations were not impacted. The youngest fully expanded leaves of 4-week old plants were put in the measurement cuvette for 10–15 min to acclimate until they reached a photosynthetic steady-state. The reference [CO_2_] was set to 400 µL L^−1^ (v/v), light intensity was 500 μmol photons m^−2^ s^−1^, temperature was 22°C, and RH was 70% to ensure that the leaf vapor pressure deficit was ∼0.85 kPa.

After the period where the leaf achieved a photosynthetic steady-state, net CO_2_ assimilation rate was logged and the gas in the cuvette was switched to a humidified (RH = 70%) mixture of 400 µL L^−1 13^CO_2_, 78% N_2_, and 21% O_2_ using three gas mass flow controllers (Alicat Scientific, Tucson AZ, USA) interfaced with a custom-programmed Raspberry Pi touchscreen monitor (Raspberry Pi foundation, code available upon request). The flow rate was initially set at 5 L min^−1^ to flush the residual ^12^CO_2_ out of the cuvette, with the atmosphere completely exchanged to ^13^CO_2_ in about 5 s as estimated by monitoring the CO_2_ concentration measured in the cuvette by the infra-red gas analyzer of the LI-COR 6800, which is less sensitive to ^13^CO_2_ as compared to ^12^CO_2._ Once the concentration of ^13^CO_2_ was constant, the flow rate of gas mixture was reduced to 1 L min^−1^ during the labeling period, which produced a similar flow rate through the sample cuvette, as before the labeling period when steady-state gas exchange was determined. Labeled leaf samples were collected at time points of 0, 0.5, 1, 2, 2.5, 3, 5, 7, 10, 15, 30, and 60 min during the labeling period, and the order in which each time point was measured was randomized across all experimental replicates. Three biological replicates for each time point were collected.

To quench metabolism at a given time point, liquid nitrogen was sprayed directly onto the leaf surface via an injection port on the cuvette after removing the adhesive putty. While liquid nitrogen sprays have been used previously to rapidly freeze leaves during gas exchange measurements (Tcherkez et al., 2012), we have applied this technique during a ^13^CO_2_ labeling experiment within the INST-MFA framework and have further quantified its quench speed. The quench speed was rapid, taking 0.1 s to reach −10°C at the leaf center and 0.5 s at the extreme leaf margin area enclosed at the edge of the gas exchange cuvette, as measured using a datalogger and a butt-end fine wire thermocouple threaded through the leaf (Supplemental Figure S6C). This system minimizes the time that leaves are exposed to transients in temperature or ambient gas concentrations (produced by the evaporation of the nitrogen gas) before metabolism is quenched following the labeling. The rapidly frozen leaf portions were then cut and placed in a 50 mL conical vial with liquid nitrogen and stored at −80°C until later mass spec analysis. Each labeling time point had three biological replicates.

To compare this improved approach with other approaches, we used a slower quenching method where the chamber was opened, and the leaves were dropped immediately into liquid nitrogen. There were substantial differences in metabolite levels measured using these slow and fast quenching approaches (Supplemental Figure S6E). The levels of RUBP, R5P, RU5P/XU5P, GAP/DHAP were lower in the slow-quenching method. The ratio of PGA/RUBP changed during slow quenching, showing that the short dark period that occurred during slow quenching altered the rate of metabolite interconversions.

### Metabolite extraction and LC-MS/MS and GC-MS analysis

Metabolites were extracted using a modification of the method described in Lunn et al. (2006). Frozen leaf tissue was ground to a fine powder and extracted with chloroform/methanol (3:7, v/v) with vigorous shaking, and incubated at −20°C for 2 h with occasional mixing. For absolute quantification of unlabeled samples, known concentrations of internal standards (D-[UL-^13^C_6_] fructose 1, 6-bisphosphate, 98 atom % ^15^N, 98 atom % ^13^C free amino acid mixture, adonitol, and [UL-^13^C_12_] sucrose) were added to the leaf tissue before extraction. Water soluble metabolites were extracted by adding 350 μL of water with vigorous shaking and centrifugation at 4,200 g for 10 min. The upper methanol-water phase was recovered and aliquoted for reverse phase LC-MS/MS, anion exchange LC-MS/MS, and GC-MS. Aliquoted extracts were evaporated to dryness using a freeze dryer and stored in −80°C before analysis.

### Reverse phase LC-MS/MS analysis

Most C_3_ cycle intermediates were analyzed by a reverse phase LC-MS/MS method. Metabolites were reconstituted in 100 μL of water from the lyophilized extract, and 10 μL of this reconstituted sample was run by an ACQUITY UPLC pump system (Waters, Milford, MA, USA) coupled with a Quattro Premier LC-MS/MS system (Waters, Milford, MA, USA) . Metabolites were separated by a 2.1 × 50 mm ACQUITY UPLC BEH C18 Column (Waters, Milford, MA, USA) at 40°C. A multi-step gradient was applied with mobile phase A (10 mM tributylamine in 5%(v/v) methanol) and mobile phase B (methanol): 0–1 min, 95%–85% A; 1–6 min, 65%–40% A; 6–7 min, 40%–0% A; 7–8 min, 0% A; 8–9 min, 100% A, at a flow rate of 0.3 mL min^−1^. Mass spectra were acquired using multiple reaction monitoring (MRM) in negative electrospray ionization (ESI) mode as described in Preiser et al. (2019) with slight modifications. The source temperature was 120°C and the desolvation temperature was 350°C. Nitrogen was used as a sheath and auxiliary gas and collision gas (argon) was set to 1.1 mTorr. Gas flows for the desolvation and cone were set to 800 and 50 L/h, respectively. The scan time was 0.1 ms. Parent-product ion transitions for metabolites were described in the Supplemental Table S2.

### Anion exchange LC-MS/MS analysis

Other phosphorylated metabolites (e.g., sugar phosphate, 2PG, and PEP) and nucleotide sugars (ADPG and UDPG) were analyzed using an anion-exchange LC-MS/MS method described in Alonso et al. (2010) with slight modifications. Metabolites were reconstituted in 100 μL of water from lyophilized extract, and 10 μL of extracts was injected into an ACQUITY UPLC pump system (Waters, Milford, MA, USA) coupled with a Xevo ACQUITY TQ Triple Quadrupole Detector (Waters, Milford, MA, USA). Metabolites were separated by an IonPac AS11 analytical column (2 × 250 mm, Dionex) equipped with an IonPac guard column AG11 (2 × 50 mm, Dionex) at a flow rate of 0.35 mL min^−1^. A multi-step gradient was applied with mobile phase A (0.5 mM KOH) and mobile phase B (75 mM KOH): 0–2 min, 100% A; 2–4 min, 100%–93% A; 4–13 min, 93%–60% A; 13–15 min, 0% A; 15–17 min, 100% A. The KOH concentration was suppressed by a post-column anion self-regenerating suppressor (Dionex ADRS 600, Thermo Scientific), with a current of 50 mA and flow rate of 3.5 mL min^−1^. An IonPac ATC-3 Anion Trap Column (4 × 35 mm), conditioned with 2M KOH, was used to remove contaminant ions from KOH solvents. Mass spectra were acquired using MRM in negative ESI mode. Parent-product ion transitions for metabolites were described in Supplemental Table S2.

### GC-MS analysis

Amino acids, organic acids, and sucrose were analyzed using a GC-MS system. Amino acids and organic acids were derivatized by methoximation, followed by tert-butyldimethylsilylation. Sucrose was derivatized by methoximation, followed by trimethylsilylation. Samples were analyzed by an Agilent 7890 GC system (Agilent, Santa Clara, CA, USA) coupled to an Agilent 5975C inert XL Mass Selective Detector (Agilent, Santa Clara, CA, USA) with an autosampler (CTC PAL; Agilent, Santa Clara, CA, USA). Metabolites were separated by an Agilent VF5ms GC column, 30 m × 0.25 mm × 0.25 m with 10 m guard column (Part number: CP9013; Agilent, Santa Clara, CA, USA). For amino acids and organic acids, 1 μL of the derivatized sample was injected into 10 split mode with helium carrier gas with a flow rate of 1.2 mL min^−1^. The oven temperature gradient was: 100°C (4 min hold), increased by 5°C/min to 200°C, then by 10°C/min to 320°C, and held at 320°C for 10 min. Electron ionization (EI) is at 70 eV and the mass scan range was 100–600 amu. The ionization source temperature was set at 150°C and the transfer line temperature at 300°C. The fragment ions used for the isotopologue analysis are described in the Supplemental Table S2.

### Analysis of mass spectrometry data

Data from LC-MS/MS were acquired with MassLynx 4.0 (Agilent, Santa Clara, CA, USA). Data from GC-MS were acquired with Agilent GC/MSD Chemstation. Metabolites were identified by mass to charge ratio (m/z), retention time in comparison with authentic standards, and the NIST library. Both LC-MS and GC-MS data were converted to MassLynx format and processed with QuanLynx software for peak detection and quantification. Metabolites were quantified either by internal standards or calibration curves obtained using external authentic standards.

### Measurement of starch, vascular sucrose, and amino acids

Starch accumulation was measured between 2 and 8 h after illumination at hourly intervals to ensure measurement at steady-state and quantify starch synthesis rate. Measurement and analysis of starch was performed by the total starch (AA/AMG) test kit (Megazyme, Bray, Wicklow, Ireland). Frozen leaf tissue was ground to a fine powder containing starch after the extraction was digested by α-amylase and amyloglucosidase. The released glucose was measured with glucose oxidase and quantified spectrophotometrically at 510 nm. The values were compared against a standard curve determined at the same time as the samples were analyzed. The export of carbon from leaves to other parts of the plant was estimated by measuring the vascular sucrose and amino acids. Fresh leaves were cut at the base and placed inside a sealed pressure bomb chamber. Pressurized gas was slowly added to the chamber until the exudates were forced out of the vasculature and visible at the cut end of the petiole. Vascular sucrose and amino acids were then collected and quantified using GC-MS as described above.

### Sucrose synthesis rate measurement and calculation

To determine the sucrose synthesis rate, sucrose labeling was determined from the mass spectra measured above. The fragments with m/z 361 and 451 represent glucosyl and fructosyl moieties of sucrose (Koubaa et al., 2012; Beshir et al., 2017). Koubaa et al. (2012) reported that m/z 361 is composed of about 40% fructosyl moiety and 60% glucosyl moiety, whereas m/z 451 is composed of 95% fructosyl moiety and 5% glucosyl moiety. We ran the authentic standard of [UL-^13^$C_{6}^{\text{fru}}$] sucrose and confirmed that m/z 361 is composed of 54% fructosyl moiety and 46% glucosyl moiety, whereas m/z 451 is composed of 92% fructosyl moiety and 8% glucosyl moiety. The MIDs of glucosyl (fGlu) and fructosyl (fFru) moieties of sucrose were calculated by the MIDs of m/z 361 (f361) and 451 (f451), and the percentage of glucosyl (46%, 8%) and fructosyl (54%, 92%) moieties in m/z 361 and 451 as:

f361=0.54*fFru+0.46*fGlu, f451=0.92*fFru+0.08fGlu.

The measured unlabeled M0 isotopologue labeling pattern suggested that there was a 60% active pool for sucrose synthesis and 40% inactive pool for sucrose export to vascular exudate. The sucrose synthesis rate was calculated assuming 60% of the active pool, the measured sucrose concentration per fresh weight, and 0.5 h for reaching the export pool of glucosyl and fructosyl moieties as according to:

$$60\%*\left[\text{Suc}\right]/0.5\text{hr}=0.6*3.6\;\mu \text{mol}\cdot {\text{gFW}}^{-1}/0.5\;\text{hr}=4.3\pm 1.08\;\mu \text{mol}\cdot {\text{gFW}}^{-1}\cdot {\text{hr}}^{-1}.$$

### G1P is excluded from the model due to the anomalous behavior observed in the G1P labeling data and minor impact to the major findings

Like others, we found anomalous behavior of the apparent G1P peak, which showed a very slow and incomplete labeling pattern with only 67% ^13^C enrichment after 60 min (Supplemental Figure S7A); this could indicate a large, metabolically inert pool, or coelution of a different hexose phosphate. While Mannose 1 phosphate coeluted with G1P, experiments reported by Szecowka et al. indicate that in Arabidopsis this is not the case. Another coeluting hexose phosphate could also be a metabolically inert pool of beta glucose-1-phosphate and metabolically separate from alpha glucose–phosphate (Belocopitow and Maréchal, 1970). Due to this uncertainty, we excluded G1P labeling kinetics from our model. To test how important this G1P data was to the model, we included G1P labeling data in the model as input data with dilution pool parameters for G1P inactive pool. Both fluxes (Supplemental Figure S8) and SSR (Supplemental Figure S9) for all models with G1P input were reported. G1P input had very limited influence for both fluxes and SSR. 94% of *R_L_* source was from the G6P/OPP shunt in the forced CO_2_.np model with constrained *v_o_/v_c_* (Supplemental Figure S8). The SSR for the forced CO_2_.np model was 926, which was smaller than the amounts calculated by the forced TCA model and the forced fatty acid model (Supplemental Figure S9).

### Amino acid biosynthesis was not included as a potential decarboxylation reaction

Amino acid biosynthesis was not included as a potential decarboxylation reaction in our model. We justify its exclusion based on several observations of published protein and amino acid turnover times. Specifically, Arabidopsis protein half-lives vary from several hours to several months (Nelson et al., 2014; Li et al., 2017). The fastest turnover of protein in barley has a *K_d_* of 1.65 d^−1^ and varies by more than 100-fold over the 508 measured proteins (Nelson et al., 2014). Abadie et al. (2020) showed protein turnover in sunflower leaves is also slow. Initial work in the Shachar-Hill lab demonstrates through D_2_O labeling that protein turnover in a camelina leaf is about 1% per day.

### Isotopomer network and flux determination

The metabolic network model with all reactions and their respective carbon atom transitions describing photosynthetic central metabolism in *C. sativa* was constructed based upon the Arabidopsis model (Ma et al., 2014) and KEGG database. A list of the reactions and abbreviations is provided in Supplemental Dataset S9. INST-MFA was performed to estimate metabolic fluxes using the Isotopomer Network Compartmental Analysis software package (INCA1.8, http://mfa.vueinnovations.com, Vanderbilt University; Young, 2014) and implemented in MATLAB 2018b. INCA uses the elementary metabolite unit (EMU) framework (Antoniewicz et al., 2007) to efficiently calculate isotopic labeling trajectories of measurable metabolites. The input of INCA includes isotopic labeling measurements, net CO_2_ assimilation rate, sucrose and starch synthesis rates, and the sucrose and amino acid concentrations in vascular exudates. Forty-one fragment ions from 27 metabolites in three replicates were analyzed by using reverse phase LC-MS/MS, ion exchange LC-MS/MS and GC-MS. Labeled isotopologues were corrected for natural abundance of labeled atoms (i.e. C) as well as unlabeled atoms introduced by derivatization or heteroatoms that were not a part of the labeling experiment (i.e. N, O, Si, S). The model has 72 free fluxes to simulate the nonlinear regression of MIDs measurements at 0, 0.5, 1, 2, 2.5, 3, 5, 7, 10, 15, 30, and 60 min. The pool of the metabolites is shown in Supplemental Table S3, although INST-MFA flux estimations are not sensitive to pool size estimations (Ma et al., 2014).

A Levenberg-Marquardt optimization algorithm was used by INCA to minimize the differences between the measured and simulated MIDs by minimizing the variance-weighted sum of squares residuals (SSRS; Levenberg, 1944; Young, 2014). The vectors of free fluxes, pool sizes, and MS scaling factors were adjusted to renormalize the measured MID vector. The smaller the SSR, the better the model fits the data. The greater the SSR, the poorer the model fits the data. The SSR of the model with no constraint to both *v_o_*/*v_c_* and *R***_*L*_** is 810 and the model fit is accepted based on χ^2^ tests with 1,753 degrees of freedom. Flux parameters were iteratively updated to converge to a best-fit solution. Individual residual errors for measurements were examined to evaluate the contribution of each measurement to the estimation of fluxes. Flux maps were drawn in a scalable vector graphic using the software Inkscape, with the best-fix ﬂux values displayed using the software FluxVisualizer (Rose and Mazat, 2018).

### Assessment of flux precision

Two independent methods were used to compute the 95% confidence intervals of the estimated flux values. An INCA built-in parameter continuation method was used to evaluate the sensitivity of the SSR to parameter variations, as described previously (Antoniewicz et al., 2006). A Monte Carlo method was also performed to determine the confidence intervals based on the uncertainty of experimental measurements. Specifically, the external flux measurements and MID data were perturbed by normally distributing noise within the measured sd. INCA was then repeatedly run using 3,000 sets of perturbed data for the calculation of flux values. The resulting distribution of flux values enabled the estimation of confidence intervals. The computation-intensive parameter continuation and Monte Carlo simulations were computed in parallel using a SLURM job scheduler to distribute jobs to hundreds of computer nodes within a high-performance computing cluster provided by the Institute for Cyber-Enabled Research at Michigan State University. The two approaches gave similar results of conﬁdence intervals for each flux solution.

## Supplemental data

**Supplemental Figure S1**. Transient ^13^CO_2_ labeling in all measured ions.

**Supplemental Figure S2**. Average ^13^C-enrichments of measured metabolites.

**Supplemental Figure S3**. Heatmap of SSR and *v_o_/v_c_* for models of unconstrained, forced CO_2_ non-photorespiratory CO_2_ release, forced TCA, and forced fatty acid with and without constraints of *v_o_/v_c_*.

**Supplemental Figure S4**. Sucrose synthesis rate calculation and the labeling of glucosyl and fructosyl moieties.

**Supplemental Figure S5**. Estimate of inactive pool contributions.

**Supplemental Figure S6**. Demonstration of the fast quenching and switching system developed for in vivo ^13^CO_2_-labeling of a single leaf.

**Supplemental Figure S7**. Incomplete labeling of G1P may result from coeluted mannose 1-phosphate.

**Supplemental Figure S8**. Heatmap for net fluxes with G1P input for models of normal, forced CO_2_ non-photorespiratory CO_2_ release, forced G6P/OPP shunt, forced TCA, and forced fatty acid.

**Supplemental Figure S9**. Heatmap of SSR with G1P input for models of unconstrained, forced CO_2_ non-photorespiratory CO_2_ release, forced TCA, and forced fatty acid with and without constraints of *v_o_/v_c_*.

**Supplemental Table S1**. Sucrose and amino acid levels and ratios of amino acids to sucrose in vascular exudates.

**Supplemental Table S2**. Parameters for transitions of measured metabolites in multiple reaction monitoring (MRM) with LC-MS/MS and selected ion monitoring (SIM) with GC-MS.

**Supplemental Table S3**. Metabolite pool sizes.

**Supplemental Dataset S1**. Estimated flux values and 95% confidence intervals by parameter continuation and Monte Carlo analysis for net and exchange fluxes for unconstrained model.

**Supplemental Dataset S2**. Estimated flux values and 95% confidence intervals by parameter continuation and Monte Carlo analysis for net and exchange fluxes for the forced CO_2_.np model.

**Supplemental Dataset S3**. Estimated flux values and 95% confidence intervals by parameter continuation and Monte Carlo analysis for net and exchange fluxes for the forced TCA model.

**Supplemental Dataset S4**. Estimated flux values and 95% confidence intervals by parameter continuation and Monte Carlo analysis for net and exchange fluxes for the forced fatty acid model.

**Supplemental Dataset S5**. Estimated flux values and 95% confidence intervals by parameter continuation and Monte Carlo analysis for net and exchange fluxes for constrained *v_o_*/*v_c_* model.

**Supplemental Dataset S6**. Estimated flux values and 95% confidence intervals by parameter continuation and Monte Carlo analysis for net and exchange fluxes for constrained *v_o_*/*v_c_* and forced CO_2_.np model.

**Supplemental Dataset S7**. Estimated flux values and 95% confidence intervals by parameter continuation and Monte Carlo analysis for net and exchange fluxes for constrained *v_o_*/*v_c_* and forced TCA model.

**Supplemental Dataset S8**. Estimated flux values and 95% confidence intervals by parameter continuation and Monte Carlo analysis for net and exchange fluxes for the constrained *v_o_*/*v_c_* and forced fatty acid model.

**Supplemental Dataset S9**. Abbreviations for metabolites and reactions.

## Supplementary Material

kiab076_Supplementary_Data

## Notes

Y.S-H. and B.J.W. conceived the original research plans; Y.S-H. and B.J.W. supervised the experiments; Y.X. performed most of the experiments; B.J.W., X.F., and T.D.S. provided technical assistance to Y.X.; Y.X. and X.F. analyzed the data; Y.X. conceived the project and wrote the article with contributions from all the authors. B.J.W. agrees to serve as the author responsible for contact and ensures communication.

The author responsible for distribution of materials integral to the findings presented in this article in accordance with the policy described in the Instructions for Authors (https://academic.oup.com/plphys/pages/general-instructions) is: Berkley J. Walker (berkley@msu.edu).
