# Supplementary material for: The metabolic origins of non-photorespiratory CO2 release during photosynthesis: a metabolic flux analysis
Source: Plant Physiol. 2021 Feb 16;186(1):297–314. doi: 10.1093/plphys/kiab076 (PMC8154043; doi:10.1093/plphys/kiab076)
Supplement: kiab076_Supplementary_Data [file kiab076_supplementary_data.zip › pp.01790.2020-s01.pdf]

Supplemental Figures

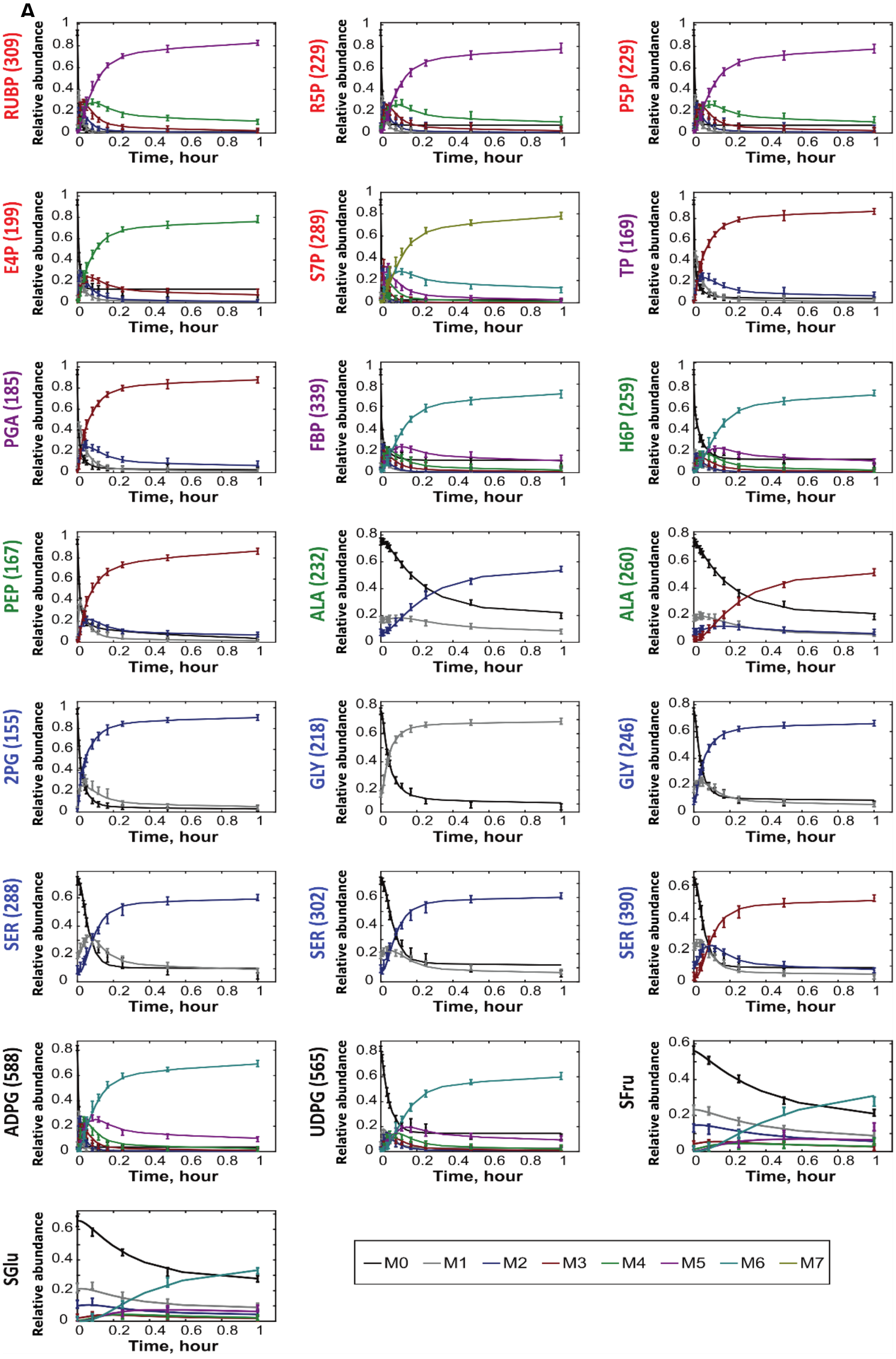

B

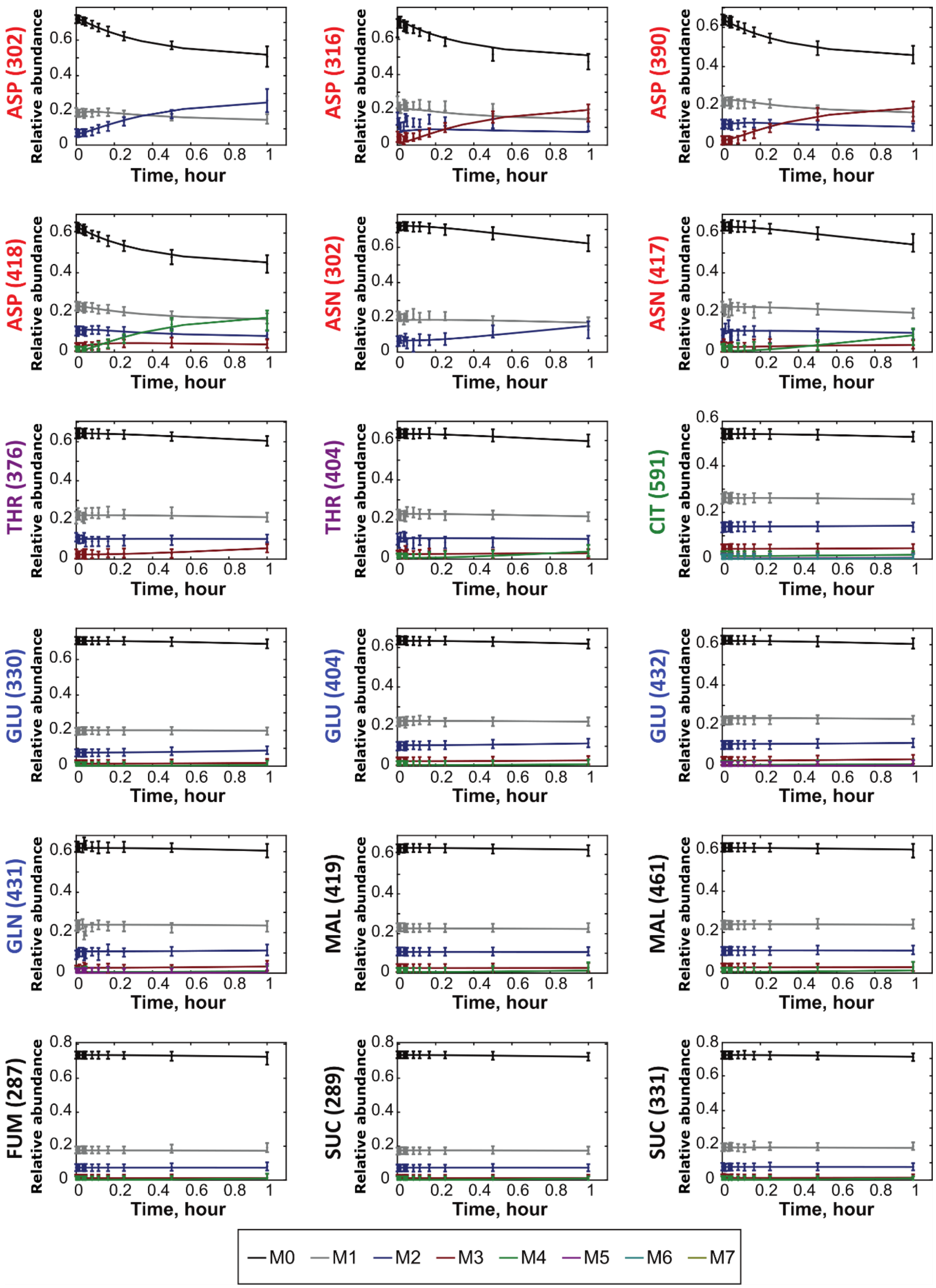

ASP (302)
ASP (316)
ASP (390)
ASP (418)
ASN (302)
ASN (417)
THR (376)
THR (404)
CIT (591)
GLU (330)
GLU (404)
GLU (432)
GLN (431)
MAL (419)
MAL (461)
FUM (287)
SUC (289)
SUC (331)
Relative abundance
Time, hour
M0
M1
M2
M3
M4
M5
M6
M7

Supplemental Figure S1. Transient $^{13}CO_2$ labeling in all measured ions.

Experimentally determined isotope labeling measurements are shown as points with error bars (n=3, ± stdev). INST-MFA fitted mass isotopologue distributions are shown as solid lines. Nominal masses of M0 mass isotopologues are shown in parentheses. The nominal mass isotopomer is represented as M0 are shown in parentheses. Other labeled isotopomers are distinguished by their mass differences from M0 (M1, M2, M3, etc.). **(A) C3 and Glycolysis related metabolites.** Core C3-only intermediates (pentose phosphates, E4P, S7P) [y-axes labeled in red]; intermediates shared with glycolysis (DHAP, PGA, FBP) [purple]; core glycolysis metabolites (G6P) and products (PEP, Ala) [green]; photorespiratory intermediates (2PG, Ser, Gly) [blue]; then carbohydrate building substrates (UDPG, ADPG, SFru, SGlu)[black]. **(B) TCA cycle related metabolites.** OAA derived AAs (Asp and Asn) [y-axes labeled in red], and more slowly, Thr [purple], which is made from Asp at a slower rate than Asn; Citrate [green]; Glu and Gln ions [labeled Glx in blue]; Malate Fumarate and Succinate [black].

A

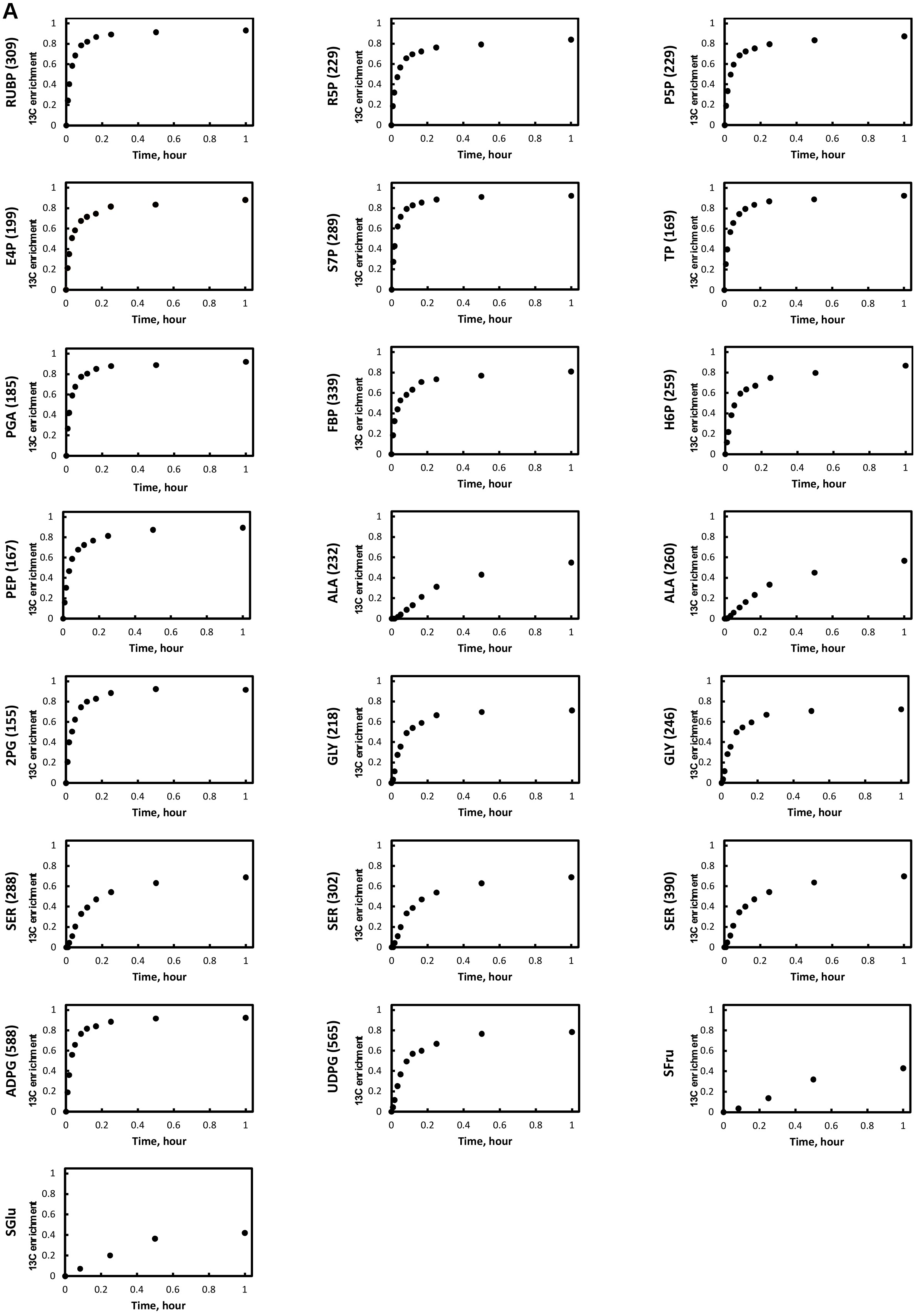

RUBP (309)
R5P (229)
P5P (229)
E4P (199)
S7P (289)
TP (169)
PGA (185)
FBP (339)
H6P (259)
PEP (167)
ALA (232)
ALA (260)
2PG (155)
GLY (218)
GLY (246)
SER (288)
SER (302)
SER (390)
ADPG (588)
UDPG (565)
SFru
SGlu
13C enrichment
Time, hour

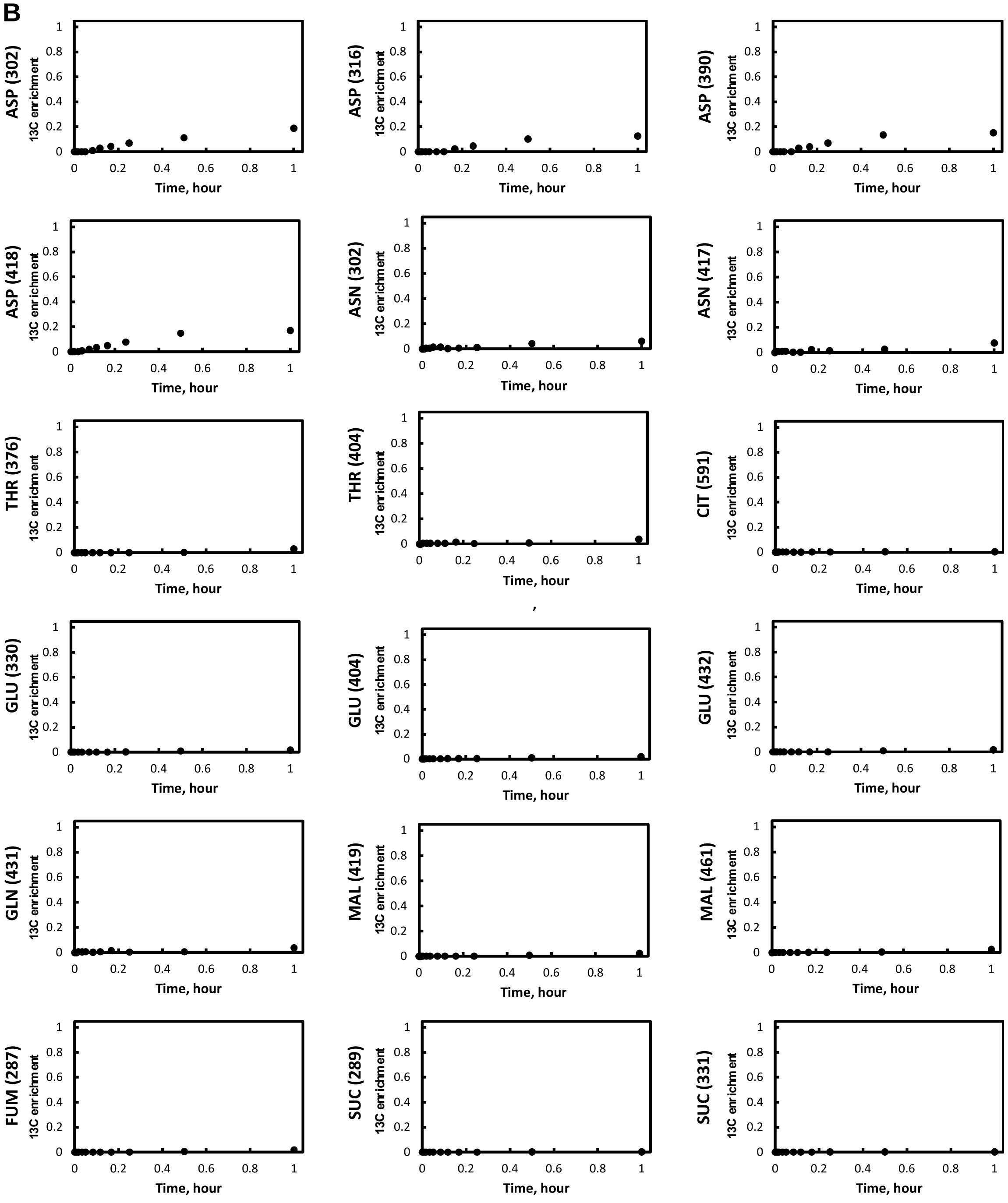

Supplemental Figure S2. Average ^{13}C-enrichments of measured metabolites.

^{13}C-enrichments were calculated by the formula of $(\frac{1}{N})\sum_{i=1}^{N} Mi \times i$, where M_i represents the N i=1 fraction of the mass isotopomer distribution (MID) after natural isotope abundance correction, and N represents the number of carbon atoms in the metabolite. Nominal masses of M0 mass isotopomers are shown in parentheses. Nominal masses of M0 mass isotopomers are shown in parentheses. Metabolites are separated into C_3- and Glycolysis-related metabolites (A) and TCA cycle related metabolites (B).

		Unconstrained v_o/v_c				Constrained v_o/v_c		
		Unconstrained	Forced CO2.np	Forced TCA	Forced Fatty Acid	Unconstrained	**Forced CO2.np**	Forced TCA
CO2	CO2_sink	0.0	0.1	0.4	0.4	0.4	**0.4**	0.4
CBC	RUBP309	13.5	13.2	30.1	15.9	14.0	**14.7**	35.7
CBC	PGA185	10.4	10.8	13.3	11.9	11.2	**12.3**	12.8
CBC	DHAP169	9.2	9.0	17.1	10.7	8.4	**8.9**	19.3
CBC	FBP339	32.0	31.8	56.0	42.1	32.4	**33.3**	67.8
CBC	F6P259	13.8	13.7	49.6	31.2	15.1	**15.3**	54.2
CBC	P5P229	60.5	60.7	79.4	66.7	61.4	**60.9**	83.0
CBC	R5P229	70.8	71.8	90.7	78.5	74.2	**73.8**	91.3
CBC	S7P289	35.3	34.9	79.1	36.0	35.7	**37.5**	84.6
CBC	G6P259	13.4	13.4	39.9	23.9	14.2	**14.9**	44.5
CBC	E4P199	45.6	44.9	52.3	47.0	44.4	**45.5**	56.5
PR	2PG155	14.9	15.5	28.4	19.1	17.5	**16.1**	17.2
PR	SER288	37.3	37.5	48.8	39.8	37.9	**37.6**	48.6
PR	SER302	36.3	36.3	34.8	38.6	35.6	**35.8**	34.7
PR	SER390	35.0	35.2	42.1	38.6	36.3	**36.5**	41.9
PR	GLY218	11.6	12.1	12.9	15.1	13.4	**14.2**	20.6
PR	GLY246	25.6	24.9	18.5	22.4	23.7	**30.9**	29.4
TCA	PEP167	18.6	18.6	38.4	21.0	20.1	**23.5**	47.3
TCA	CIT591	0.6	0.6	24.6	0.6	0.6	**0.6**	26.9
TCA	SUC289	0.7	0.6	0.6	0.6	0.7	**0.7**	0.5
TCA	SUC331	1.2	1.2	1.0	1.2	1.2	**1.2**	1.0
TCA	FUM287	1.1	1.1	1.1	1.1	1.1	**1.1**	1.1
TCA	MAL419	1.0	1.0	1.1	1.1	1.0	**1.0**	1.2
TCA	MAL461	1.1	1.1	1.3	1.2	1.1	**1.1**	1.3
AA	ALA232	5.2	5.3	8.0	6.4	5.9	**6.3**	7.6
AA	ALA260	7.2	7.3	10.1	8.1	7.7	**7.9**	10.0
AA	ASP302	5.2	5.2	5.2	2.8	5.1	**4.8**	5.1
AA	ASP316	64.6	64.5	78.3	65.7	63.3	**64.0**	76.0
AA	ASP390	6.8	6.6	28.0	4.9	7.7	**5.8**	28.4
AA	ASP418	5.2	5.0	23.3	2.6	5.5	**4.0**	23.4
AA	THR376	4.7	4.6	5.6	4.5	4.7	**4.6**	5.6
AA	THR404	7.8	7.8	8.7	7.6	7.9	**7.8**	8.7
AA	GLU330	0.7	0.7	0.7	0.7	0.7	**0.7**	0.7
AA	GLU404	1.2	1.2	1.2	1.2	1.2	**1.2**	1.2
AA	ASN302	9.2	9.2	9.3	9.3	9.2	**9.2**	9.3
AA	ASN417	11.0	10.9	13.4	10.6	11.0	**10.8**	13.4
AA	GLN431	18.0	18.0	18.0	18.0	18.0	**18.0**	18.0
AA	GLU432	4.4	4.4	4.5	4.4	4.4	**4.4**	4.5
Sucrose	UDPG565	51.6	52.1	84.5	70.5	54.0	**53.6**	84.4
Sucrose	SGlc	27.4	27.3	36.5	25.2	27.5	**27.7**	36.7
Sucrose	SFru	22.2	22.3	18.7	22.5	22.1	**22.0**	19.0
Sucrose	Sucrose	0.0	0.0	1.4	0.0	0.7	**0.7**	1.4
Starch	ADPG588	61.0	61.5	83.9	60.8	61.6	**61.6**	84.8
Starch	Starch	0.0	0.0	0.5	0.0	0.6	**0.7**	0.8
	**Total SSR**	**803**	**804**	**1202**	**891**	**820**	**834**	**1261**
	**v_o (µmol CO_2 g^{-1} FW hr^{-1})**	**12**	**17**	**16**	**23**	**31**	**31**	**31**
	**v_c (µmol CO_2 g^{-1} FW hr^{-1})**	**135**	**133**	**147**	**150**	**150**	**155**	**155**
	**v_o/v_c**	**0.09**	**0.13**	**0.11**	**0.15**	**0.20**	**0.20**	**0.20**

Highest value

50th percentile

Lowest value

Supplemental Figure S3. Heatmap of SSR and v_o/v_c for models of unconstrained, forced CO_2 non-photorespiratory CO_2 release, forced TCA, and forced fatty acid with and without constraints of v_o/v_c.

Global best fit sum-of-squared residuals (SSR) and individual squared residuals (SRES) of the best-fit flux solution were calculated by parameter continuation analysis and shown in heatmap. Four possible scenarios were tested by forcing the non-photorespiratory CO2 to equal the measured R_L: 1) unconstrained; 2) a mix of the TCA cycle, fatty acid synthesis and the G6P/OPPP shunt explained R_L (Forced CO2.np); 3) The TCA cycle alone caused R_L (Forced TCA); 4) Fatty acid synthesis alone caused R_L (Forced fatty acid). These scenarios were simulated with and without the constraint v_o/v_c in the range of 0.2 to 0.25. SSR, v_o, v_c, and v_o/v_c were shown in bold font and bigger-sized fonts. SRES for the model of forced CO_2.np with constraint v_o/v_c as shown in Figure 5 were shown in bold in box. All abbreviations are shown in Supplemental Dataset S9.

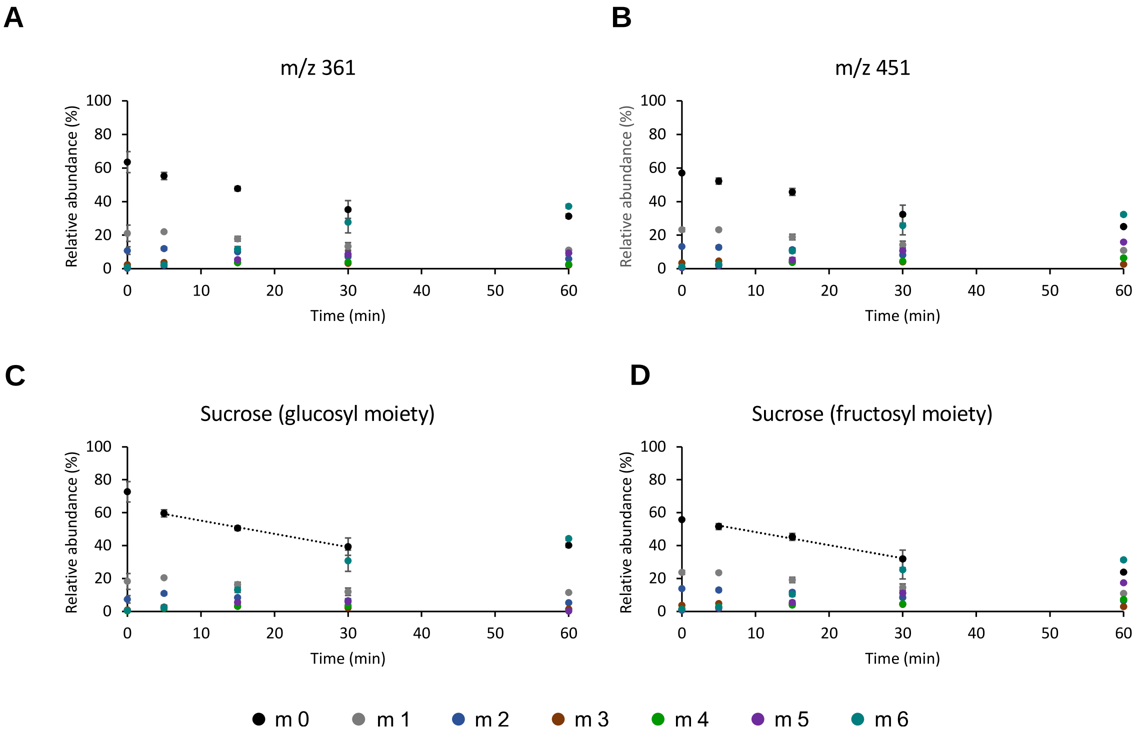

Supplemental Figure S4. Sucrose synthesis rate calculation and the labeling of glucosyl and fructosyl moieties.

The time course of mass isotopomer distribution of glucosyl (C) and fructosyl moieties (D) of sucrose were calculated based on the m/z 361(A) and 451(B) after correction with the ratios of glucosyl and fructosyl moieties in two peaks. The ratios of glucosyl and fructosyl moieties in m/z 361 and 451 were calculated based on the ratios of glucosyl and fructosyl moieties in [UL-$^{13}C_6^{fru}$] Sucrose. Fructosyl moiety of sucrose contributes about 54% to the intensity of m/z 361, and 92% to the intensity of m/z 451. Sucrose synthesis rates were calculated by using the active pool fraction (~60%), the sucrose concentration in leaf, and the sucrose turnover rate (0.5h) (n=3, ± stdev). The dotted lines represent the linear labeling of the glucosyl and fructosyl moieties of the sucrose fragments within the first 30 min.

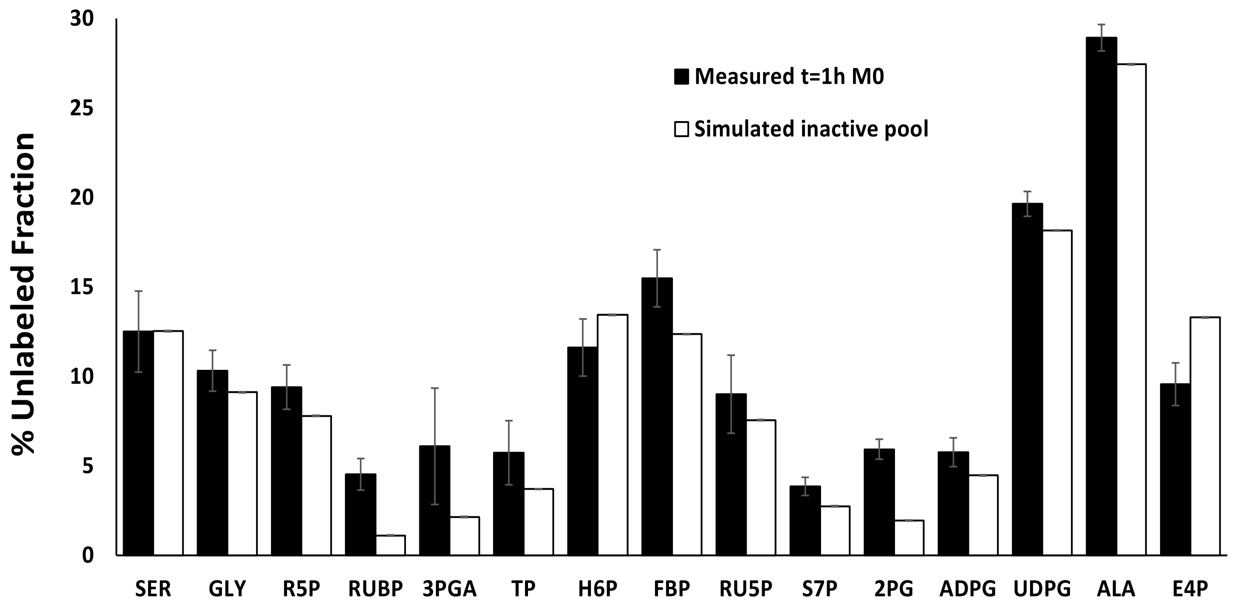

Supplemental Figure S5. Estimate of inactive pool contributions.

The experimentally measured and INST-MFA estimated unlabeled fraction at 60 min showed qualitatively similar values for most metabolites (n=3, ± stdev). C_3 cycle and photorespiration intermediates showed relatively low M0 isotopomer fractions whereas UDPG and alanine showed relatively high M0 isotopomer fractions.

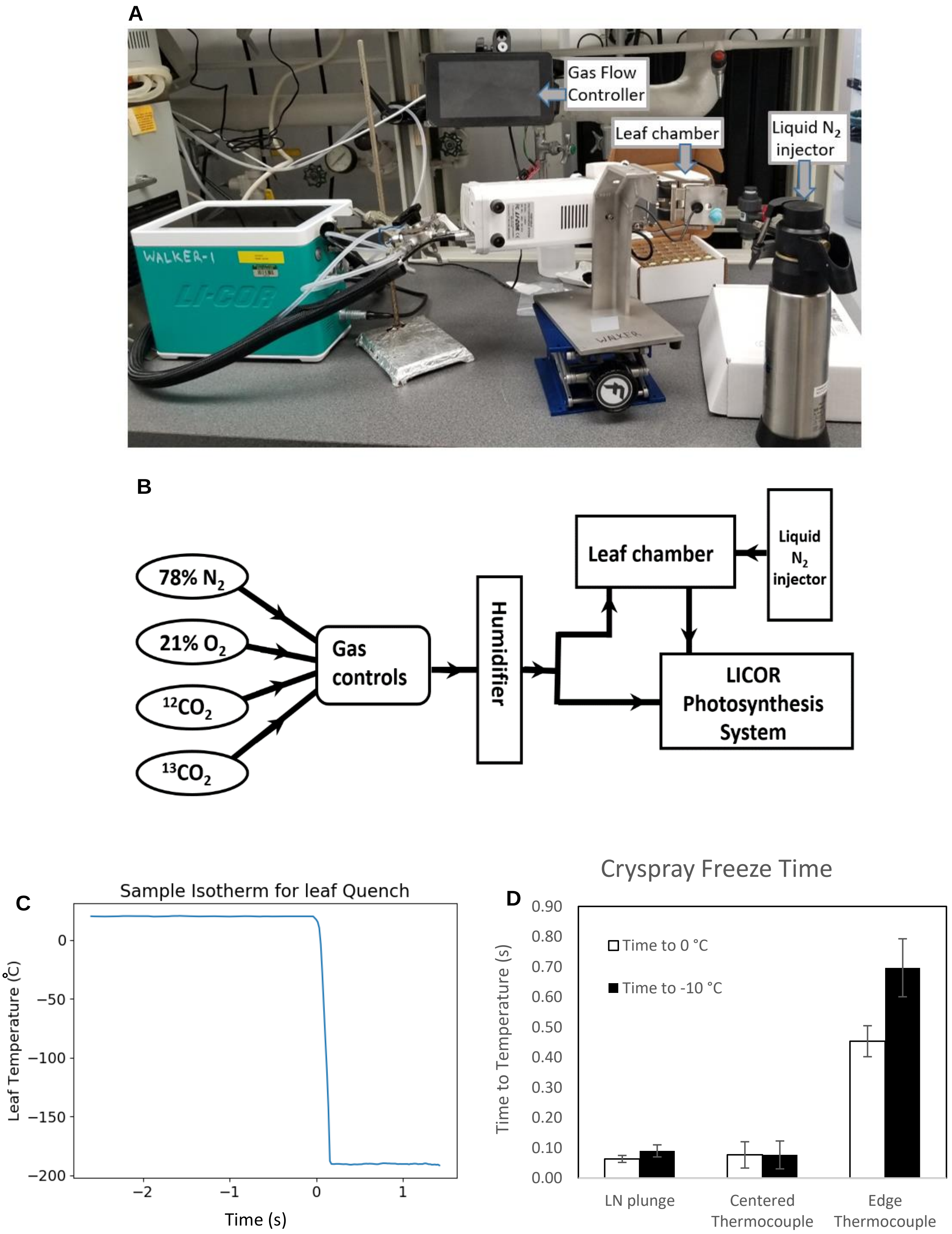
A
Gas Flow Controller
Leaf chamber
Liquid N_2 injector
B
78% N_2
21% O_2
$^{12}CO_2$
$^{13}CO_2$
Gas controls
Humidifier
Leaf chamber
Liquid N_2 injector
LICOR Photosynthesis System
C
Sample Isotherm for leaf Quench
Leaf Temperature (°C)
Time (s)
0
−50
−100
−150
−200
−2
−1
0
1
D
Cryspray Freeze Time
Time to Temperature (s)
Time to 0 °C
Time to -10 °C
0.00
0.10
0.20
0.30
0.40
0.50
0.60
0.70
0.80
0.90
LN plunge
Centered Thermocouple
Edge Thermocouple

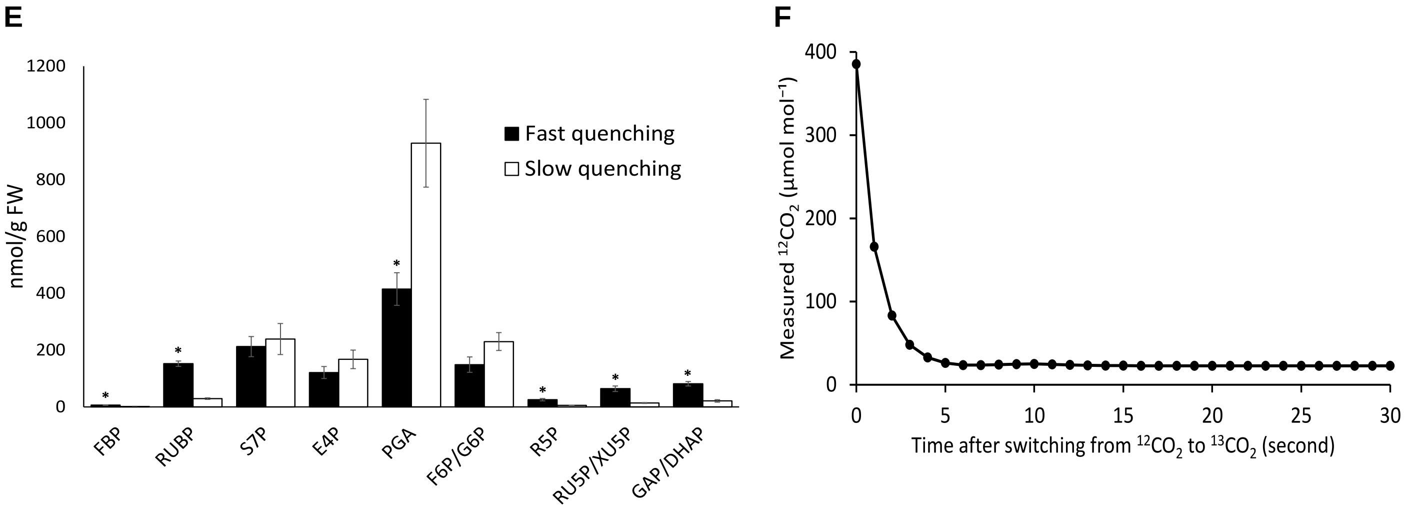

Supplemental Figure S6. Demonstration of the fast quenching and switching system developed for *in vivo* $^{13}CO_2$-labelling of single leaf. The system was composed of a LI-6800 portable photosynthesis system, an electronic gas controller, a custom modified leaf chamber, and a liquid N_2 sprayer (A), with accompanying flow diagram (B). A sample isotherm during leaf quenching was measured by sewing a butt-end thermocouple into adaxial side of a leaf (cryospray was applied to the adaxial side) and measuring the leaf temperature (blue line) during the quench from the portion of the leaf centered on the cryospray (C). Also shown is the average time it took the thermocouple to reach 0 or -10°C when plunged directly into liquid nitrogen (LN plunge), when arranged to be in the center of the chamber during the quench (Centered Thermocouple), or placed at the furthermost edge of the chamber to capture the slowest possible quench (D). The time taken to reach minimum temperature are shown. Each bar presents mean ± SD (n=3). A comparison of C_3 cycle intermediates concentrations with slow and fast quenching sampling illustrates the importance of fast quenching (n=3, ± stdev). * indicates statistically significant difference between samples with slow and fast quenching ($P \leq 0.05$, t-tests) (E). The speed of switching from $^{12}CO_2$ to $^{13}CO_2$ was monitored during each labeling time course exploiting the $^{13}CO_2$ insensitivity of the LI-COR 6800, with a representative graph shown (F).

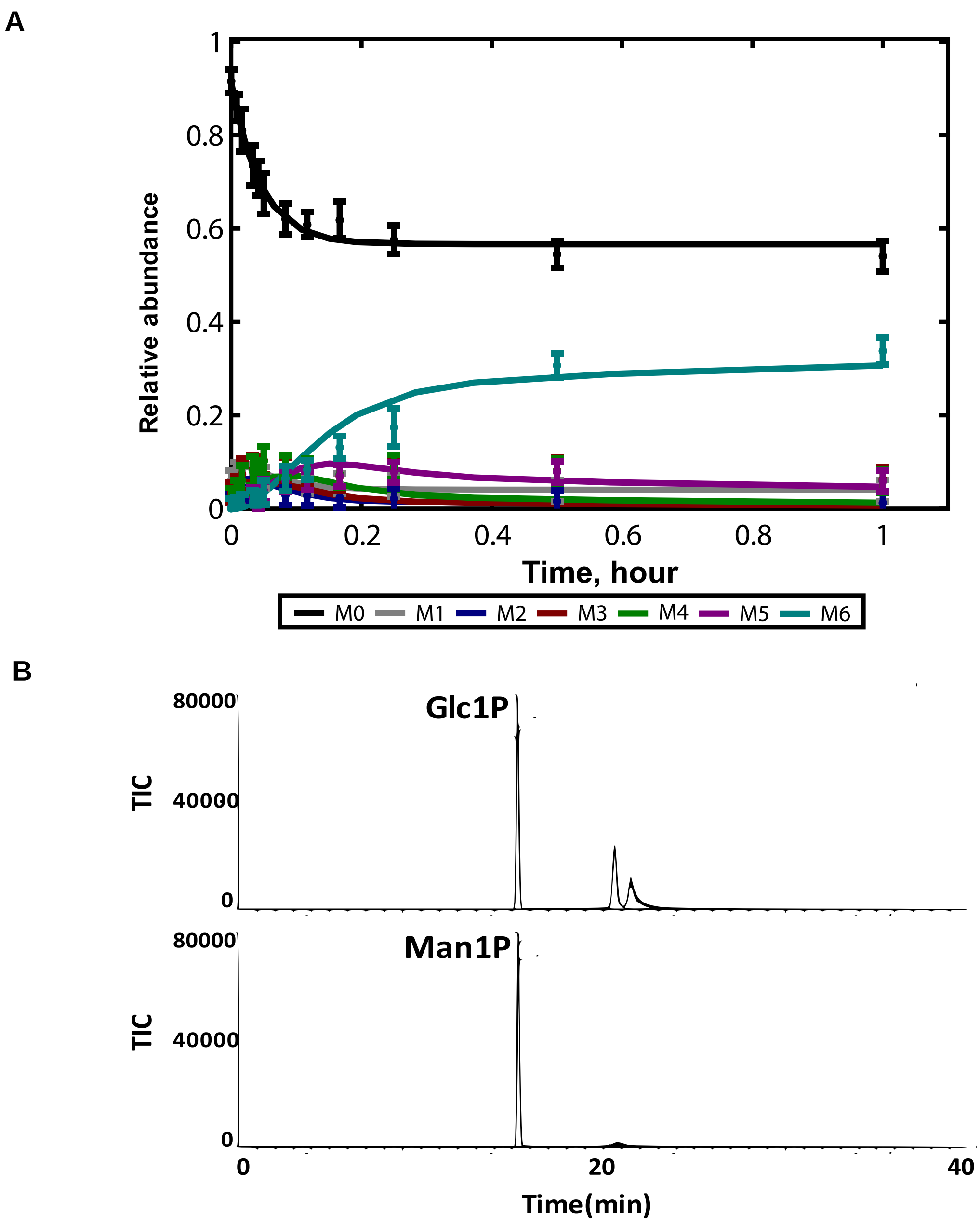

Supplemental Figure S7. Incomplete labeling of G1P may result from coeluted mannose 1-phosphate. (A) Transient $^{13}CO_2$ labeling in G1P. Experimentally determined isotope labeling measurements are shown as points with error bars (n=3, ± stdev). The nominal mass isotopomer are represented as M0. Other labeled isotopomers are distinguished by their mass differences from M0 (M1, M2, M3, etc.). INST-MFA fitted mass isotopomer distributions are shown as solid lines. **(B) Coelution of G1P and M1P in liquid chromatography.** Chromatograms of Glc1P (top graph) and Man1P (bottom graph) by LC- MS/MS. 5 mM Glc1P and Man1P standards were monitored by LC-MS/MS at the MRM 259/97. LC-MS/MS method and data analysis were described in methods. TIC: total Ion count.

Pathway	Reaction	Unconstrained v_o/v_c				Constrained v_o/v_c > 0.2			
		Unconstrained	Forced CO2.np	Forced TCA	Forced Fatty Acid	Unconstrained	Forced CO2.np	Forced TCA	Forced Fatty Acid
Non-photorespiratory CO_2 (CO_2.np)	**CO_2.np** -> CO2	12.2	9.3	9.3	9.3	5.3	**9.3**	9.3	9.3
G6P/OPPP Shunt	G6P.p -> 6PG.p	10.7	8.3	0.9	0.0	4.6	**8.7**	0.9	0.0
	6PG.p -> RU5P.p + **CO_2.np**	10.7	8.3	0.9	0.0	4.6	**8.7**	0.9	0.0
TCA cycle	3PGA.c <-> PEP.c	2.0	1.8	5.6	1.7	2.0	**2.0**	5.6	1.6
	PEP.c -> PYR.c	1.1	0.9	3.9	0.9	0.9	**0.9**	3.9	0.7
	PYR.c -> ACA.c + **CO_2.np**	1.1	0.9	3.8	0.8773	0.8	**0.8**	3.8	0.7
	OAA + ACA.c -> CIT	1.1	0.9	3.8	0.8773	0.8	**0.8**	3.8	0.7
	CIT <-> ICI	1.1	0.9	3.8	0.9	0.8	**0.8**	3.8	0.7
	ICI -> AKG + **CO_2.np**	1.1	0.9	3.8	0.9	0.8	**0.8**	3.8	0.7
	MAL <-> OAA	0.3	0.2	2.5	0.3	0.0	**0.0**	2.5	0.0
	AKG -> SUC + **CO_2.np**	0.3	0.2	2.5	0.3	0.0	**0.0**	2.5	0.0
	SUC <-> FUM	0.3	0.2	2.5	0.3	0.0	**0.0**	2.5	0.0
	FUM <-> MAL	0.3	0.2	2.5	0.3	0.0	**0.0**	2.5	0.0
Fatty Acid Synthesis	3PGA.p <-> PEP.p	0.0	0.0	0.0	8.1	0.0	**0.0**	0.0	8.8
	PEP.p <-> PYR.p	0.0	0.0	0.0	8.1	0.0	**0.0**	0.0	8.8
	PYR.p -> ACA.p + **CO_2.np**	0.0	0.0	0.0	8.1	0.0	**0.0**	0.0	8.8
	ACA.p -> fattyacid	0.0	0.0	0.0	8.1	0.0	**0.0**	0.0	8.8
Net CO_2 Uptake	CO_2.t -> CO_2	117.1	115.2	130.0	122.3	130.0	**130.0**	130.0	130.0
C3 Cycle	RUBP.p + CO_2 -> 3PGA.p + 3PGA.p	135.3	133.0	145.4	143.2	150.5	**154.8**	154.8	154.8
	3PGA.p -> TP.p	286.4	289.4	303.1	311.1	344.7	**354.0**	350.3	345.6
	TP.p + E4P.p -> SBP	45.5	47.2	52.2	55.4	58.8	**59.0**	61.6	61.9
	SBP -> S7P.p	45.5	47.2	52.2	55.4	58.8	**59.0**	61.6	61.9
	TP.p + TP.p <-> FBP.p	65.8	65.1	56.7	64.8	73.7	**78.0**	66.4	71.5
	FBP.p <-> F6P.p	65.8	65.1	56.7	64.8	73.7	**78.0**	66.4	71.5
	TP.p + EC2 <-> X5P.p	91.0	94.4	104.4	110.8	117.6	**118.1**	123.3	123.9
	S7P.p <-> R5P.p + EC2	45.5	47.2	52.2	55.4	58.8	**59.0**	61.6	61.9
	F6P.p <-> E4P.p + EC2	45.5	47.2	52.2	55.4	58.8	**59.0**	61.6	61.9
	X5P.p <-> RU5P.p	91.0	94.4	104.4	110.8	117.6	**118.1**	123.3	123.9
	R5P.p <-> RU5P.p	45.5	47.2	52.2	55.4	58.8	**59.0**	61.6	61.9
	RU5P.p -> RUBP.p	147.3	149.9	157.5	166.2	181.0	**185.8**	185.8	185.8
Photorespiration	RUBP.p -> 3PGA.p + 2PG.p	12.0	16.9	12.1	23.1	30.5	**31.0**	31.0	31.0
	2PG.p -> GLY.p	12.0	16.9	12.1	23.1	30.5	**31.0**	31.0	31.0
	GLY.p + GLY.p -> SER.p + CO_2	6.0	8.5	6.1	11.5	15.2	**15.5**	15.5	15.5
	SER.p -> GA.p	5.9	8.4	5.9	11.4	15.1	**15.4**	15.3	15.4
	GA.p <-> 3PGA.p	5.9	8.4	5.9	11.4	15.1	**15.4**	15.3	15.4
Starch Synthesis	F6P.p <-> G6P.p	20.3	17.9	4.5	9.4	15.0	**19.0**	4.7	9.5
	G6P.p <-> G1P.p	9.6	9.6	3.5	9.4	10.3	**10.3**	3.8	9.5
	G1P.p -> ADPG.p	9.6	9.6	3.5	9.4	10.3	**10.3**	3.8	9.5
	ADPG.p -> Starch	9.6	9.6	3.5	9.4	10.3	**10.3**	3.8	9.5
Sucrose Synthesis	TP.c + TP.c <-> FBP.c	9.1	8.8	16.6	7.6	10.4	**10.4**	16.4	8.4
	FBP.c <-> F6P.c	9.1	8.8	16.6	7.6	10.4	**10.4**	16.4	8.4
	F6P.c <-> G6P.c	4.5	4.4	8.3	3.8	5.2	**5.2**	8.2	4.2
	G6P.c <-> G1P.c	4.5	4.4	8.3	3.8	5.2	**5.2**	8.2	4.2
	G1P.c <-> UDPG.c	4.5	4.4	8.3	3.8	5.2	**5.2**	8.2	4.2
	UDPG.c <-> SGlc.c	4.5	4.4	8.3	3.8	5.2	**5.2**	8.2	4.2
	F6P.c <-> SFrc.c	4.5	4.4	8.3	3.8	5.2	**5.2**	8.2	4.2
	SGlc.c + SFrc.c -> S6P	4.5	4.4	8.3	3.8	5.2	**5.2**	8.2	4.2
Amino Acids	PYR.c -> ALA.c	0.1	0.1	0.1	0.0	0.1	**0.1**	0.1	0.0
	OAA -> ASP	0.2	0.2	0.4	0.2	0.2	**0.2**	0.3	0.2
	AKG -> GLU	0.7	0.7	1.3	0.6	0.8	**0.8**	1.3	0.7
	GLU <-> PRO	0.0	0.0	0.0	0.0	0.0	**0.0**	0.0	0.0
	GLU <-> GLN	0.0	0.0	0.0	0.0	0.0	**0.0**	0.0	0.0
	ASP <-> THR	0.0	0.0	0.0	0.0	0.0	**0.0**	0.0	0.0
	ASP <-> ASN	0.0	0.0	0.0	0.0	0.0	**0.0**	0.0	0.0
Transport in vascular exudates	S6P	4.5	4.4	8.3	3.8	5.2	**5.2**	8.2	4.2
	GLU	0.7	0.7	1.3	0.6	0.8	**0.8**	1.3	0.7
	ASP	0.2	0.2	0.4	0.2	0.2	**0.2**	0.3	0.2
	ALA	0.1	0.1	0.1	0.0	0.1	**0.1**	0.1	0.0
	GLY	0.0	0.0	0.0	0.0	0.0	**0.0**	0.0	0.0
	SER	0.1	0.1	0.2	0.1	0.1	**0.1**	0.2	0.1
Anaplerotic	PEP.c + CO_2 -> OAA	0.9	0.9	1.7	0.8	1.1	**1.1**	1.7	0.9
Transporters	3PGA.p <-> 3PGA.c	2.0	1.8	5.6	1.7	2.0	**2.0**	5.6	1.6
	TP.p <-> TP.c	18.1	17.6	33.3	15.3	20.8	**20.8**	32.7	16.9

Highest value

50th percentil

Lowest value

Supplemental Figure S8. Heatmap for net fluxes with G1P input for models of normal, forced CO_2 non-photorespiratory CO_2 release, forced G6P/OPP shunt, forced TCA, and forced fatty acid with and without constraints of v_o/v_c. Net fluxes were determined by ^{13}C INST-MFA by INCA using the metabolic network model and experimental inputs including mass isotopomer distributions of measured metabolites, net CO_2 assimilation, starch synthesis rate, and sucrose synthesis rate. Flux unit was expressed by μmol metabolite·gFW^{-1}·hr^{-1}. Four possible scenarios, with and without the constraint of v_o/v_c, were tested by forcing the non-photorespiratory CO_2 to equal the measured R_L: 1) a mix of the TCA cycle, fatty acid synthesis and the G6P/OPPP shunt explained R_L (Forced CO_2.np); 2) G6P/OPPP shunt alone caused R_L (Forced TCA); 3) The TCA cycle alone caused R_L (Forced TCA); 4) Fatty acid synthesis alone caused R_L (Forced fatty acid). v_c, biochemical velocity of rates of rubisco carboxylation; v_o, biochemical velocity of rates of rubisco oxygenation; constraint v_o/v_c was in the range of 0.2 to 0.25. The fluxes for the model of forced CO_2.np with constraint v_o/v_c as shown in Figure 5 were shown in bold. Sucrose and amino acid transport fluxes in vascular exudates were calculated by measured sucrose and amino acid levels in vascular exudates with the estimated flux of S6P + 0.1618*GLU + 0.0425*ASP + 0.01137*ALA.c + 0.003443*dummyGLY + 0.01997*dummySER -> Sink. All abbreviations are shown in Supplemental Dataset S9.

		Unconstrained v_o/v_c				Constrained v_o/v_c>0.2			
		Unconstrained	Forced CO2.np	Forced TCA	Forced Fatty Acid	Unconstrained	**Forced CO2.np**	Forced TCA	Forced Fatty Acid
CO2	CO2_sink	0.0	0.1	0.4	0.0	0.4	**0.4**	0.4	0.4
CBC	RUBP309	14.3	14.1	28.9	14.2	14.6	**14.9**	30.1	16.4
CBC	PGA185	10.7	10.8	12.9	11.7	11.3	**12.0**	12.5	12.0
CBC	DHAP169	8.3	8.1	13.3	10.2	8.1	**8.3**	12.7	9.6
CBC	FBP339	30.5	30.3	37.8	29.0	30.4	**33.9**	40.7	30.6
CBC	F6P259	15.3	15.1	31.1	16.2	15.8	**16.0**	32.8	17.6
CBC	P5P229	59.5	59.8	76.6	62.5	60.7	**60.9**	80.1	61.4
CBC	R5P229	69.7	70.6	88.3	75.7	73.3	**73.3**	90.5	72.8
CBC	S7P289	35.8	35.5	68.6	36.4	36.2	**37.7**	72.6	40.0
CBC	G6P259	15.1	14.9	28.0	14.6	14.2	**15.3**	29.4	15.3
CBC	E4P199	46.0	45.3	50.9	45.4	44.4	**45.7**	53.2	54.0
PR	2PG155	14.8	15.3	29.5	18.9	17.7	**16.4**	17.3	15.1
PR	SER288	37.5	37.7	48.6	38.4	37.9	**37.7**	49.6	38.0
PR	SER302	36.3	36.3	34.7	36.5	35.5	**35.8**	35.5	36.2
PR	SER390	35.3	35.4	42.2	36.3	36.3	**36.5**	42.8	36.8
PR	GLY218	11.7	12.2	12.9	13.6	13.3	**14.2**	18.3	14.5
PR	GLY246	25.8	24.9	18.2	20.5	23.5	**30.9**	27.3	23.2
TCA	PEP167	18.4	18.5	27.5	23.8	20.2	**23.2**	32.5	29.0
TCA	CIT591	0.6	0.6	2.3	0.6	0.6	**0.6**	2.3	0.6
TCA	SUC289	0.7	0.7	0.6	0.7	0.7	**0.7**	0.6	0.6
TCA	SUC331	1.2	1.2	1.1	1.2	1.2	**1.2**	1.1	1.2
TCA	FUM287	1.1	1.1	1.2	1.1	1.1	**1.1**	1.2	1.1
TCA	MAL419	1.0	1.0	1.1	1.0	1.0	**1.0**	1.1	1.0
TCA	MAL461	1.1	1.1	1.3	1.1	1.1	**1.1**	1.2	1.1
AA	ALA232	5.3	5.4	12.6	5.5	5.9	**6.3**	7.0	5.6
AA	ALA260	7.2	7.3	16.1	7.1	7.6	**7.9**	9.4	6.9
AA	ASP302	5.3	5.2	5.7	5.0	5.1	**4.8**	5.4	4.4
AA	ASP316	64.7	64.6	78.5	64.8	63.2	**63.9**	73.5	65.9
AA	ASP390	6.6	6.4	28.1	5.6	7.7	**5.8**	25.9	4.4
AA	ASP418	5.1	4.9	24.0	4.1	5.5	**4.0**	21.4	2.8
AA	THR376	4.7	4.6	5.6	4.6	4.7	**4.6**	5.5	4.6
AA	THR404	7.8	7.8	8.8	7.8	7.9	**7.8**	8.6	7.7
AA	GLU330	0.7	0.7	1.7	0.7	0.7	**0.7**	1.7	0.7
AA	GLU404	1.2	1.2	2.0	1.2	1.2	**1.2**	2.0	1.2
AA	ASN302	9.2	9.2	9.2	9.2	9.2	**9.2**	9.2	9.3
AA	ASN417	11.0	11.0	13.4	10.8	11.0	**10.8**	13.1	10.6
AA	GLN431	18.0	18.0	18.0	18.0	18.0	**18.0**	18.0	18.0
AA	GLU432	4.4	4.4	5.4	4.4	4.4	**4.4**	5.4	4.4
Sucrose	UDPG565	55.9	56.7	67.8	55.7	55.9	**56.2**	70.3	55.7
Sucrose	SGlc	28.5	28.4	37.5	28.6	28.0	**28.2**	37.6	28.4
Sucrose	SFru	22.1	22.0	18.8	22.1	22.0	**21.8**	18.7	21.9
Sucrose	Sucrose	0.0	0.0	13.7	0.2	0.7	**0.7**	12.8	0.0
Starch	ADPG588	62.4	62.9	80.4	62.0	62.7	**62.2**	76.3	100.9
Starch	Starch	0.0	0.0	39.4	0.0	0.7	**0.7**	35.4	0.0
**Sucrose/Starch**	**G1P259**	**76.1**	**75.8**	**81.4**	**76.0**	**76.5**	**88.3**	**83.4**	**76.3**
	**Total SSR**	**887**	**887**	**1227**	**903**	**898**	**926**	**1226**	**958**
	**v_o (µmol CO_2 g^{-1} FW hr^{-1})**	**12**	**17**	**12**	**23**	**31**	**31**	**31**	**31**
	**v_c (µmol CO_2 g^{-1} FW hr^{-1})**	**135**	**133**	**145**	**143**	**150**	**155**	**155**	**155**
	vo/vc	**0.09**	**0.13**	**0.08**	**0.16**	**0.20**	**0.20**	**0.20**	**0.20**

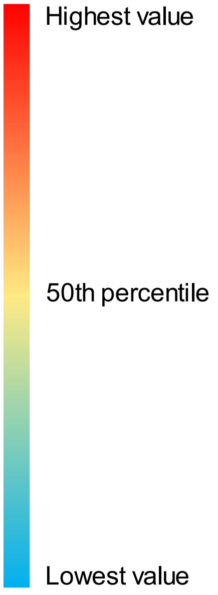

Supplemental Figure S9. Heatmap of SSR with G1P input for models of unconstrained, forced CO_2 non-photorespiratory CO_2 release, forced TCA, and forced fatty acid with and without constraints of v_o/v_c. Global best fit sum-of-squared residuals (SSR) and individual squared residuals (SRES) of the best-fit flux solution were calculated by parameter continuation analysis and shown in heatmap. Four possible scenarios were tested by forcing the non-photorespiratory CO2 to equal the measured R_L: 1) unconstrained; 2) a mix of the TCA cycle, fatty acid synthesis and the G6P/OPPP shunt explained R_L (Forced CO2.np); 3) The TCA cycle alone caused R_L (Forced TCA); 4) Fatty acid synthesis alone caused R_L (Forced fatty acid). These scenarios were simulated with and without the constraint v_o/v_c in the range of 0.2 to 0.25. SSR, v_o, v_c, and v_o/v_c were shown in bold font and bigger-sized fonts. SRES for G1P and the model of forced CO_2.np with constraint v_o/v_c as shown in Figure 5 were shown in bold in box. All abbreviations are shown in Supplemental Dataset S9.

Supplemental Tables

Supplemental Table S1. Sucrose and amino acid levels and ratios of amino acids to sucrose in vascular exudates. The levels of sucrose and several amino acids were in vascular exudates were measured by cutting fresh rosettes and extract the exudates with a pressure bump to encourage pressure release of liquid. The ratios of sucrose to several amino acids (glutamine (Gln), glutamate (Glu), proline (Pro), asparagine (Asn), aspartate (Asp), threonine (Thr), alanine (Ala), serine (Ser), and glycine (Gly) were used to constrain the output flux reaction (S6P + 0.1618*GLU + 0.0425*ASP + 0.01137*ALA.c + 0.003443*dummyGLY + 0.01997*dummySER -> Sink) to accurately depict the noncyclic TCA pathway activity.

Parameter Type	Metabolite or ratio	Concentration or ratio
Levels (nmol/ul=mM)	Sucrose	9.18 ± 1.59
	Glu	1.49 ± 0.20
	Asp	0.39 ± 0.05
	Ala	0.10 ± 0.02
	Gly	0.03 ± 0.003
	Ser	0.18 ± 0.03
Ratio (nmol/nmol)	Glu/Sucrose	0.1618
	Ala/Sucrose	0.0425
	Ala/Sucrose	0.0114
	Gly/Sucrose	0.0034
	Ser/Sucrose	0.0200

Supplemental Table S2. Parameters for transitions of measured metabolites in multiple reaction monitoring (MRM) with LC-MS/MS and selected ion monitoring (SIM) with GC-MS. LC-MS/MS dwell time was set at 20 ms for each transition. Q1, m/z of the precursor ion; Q3, m/z of the product ion. Cone and collision energy were optimized by direct infusion of standards. Amino and organic acids were measured by GC-MS by tert-butyldimethylsilyl (TBDMS) derivatization whereas sucrose was derivatized by trimethylsilyl (TMS).

Multiple reaction monitoring (MRM) with LC-MS/MS

Metabolites	C atoms	Isotopomers	$Q_1[Q_3]$ (m/z) of mass isotopomers	Cone (V)	Collision (V)
**PGA**	**3**	$[M]^+$ - $[M+3]^+$	185[97], 186[97], 187[97], 188[97]	16	17
**GAP/DHAP**	**3**	$[M]^+$ - $[M+3]^+$	169[97], 170[97], 171[97], 172[97]	18	10
**E4P**	**4**	$[M]^+$ - $[M+4]^+$	199[79], 200[79], 201[79], 202[79], 203[79]	34	13
**P5P**	**5**	$[M]^+$ - $[M+5]^+$	229[97], 230[97], 231[97], 232[97], 233[97], 234[97]	16	17
**RUBP**	**5**	$[M]^+$ - $[M+5]^+$	309[97], 310[97], 311[97], 312[97], 313[97], 314[97]	28	21
**FBP**	**6**	$[M]^+$ - $[M+6]^+$	339[97], 340[97], 341[97], 342[97], 343[97], 344[97], 345[97]	16	25
**HP**	**6**	$[M]^+$ - $[M+6]^+$	259[97], 260[97], 261[97], 262[97], 263[97], 264[97], 265[97]	26	18
**S7P**	**7**	$[M]^+$ - $[M+7]^+$	289[97], 290[97], 291[97], 292[97], 293[97], 294[97], 295[97], 296[97]	20	20
**G1P**	**6**	$[M]^+$ - $[M+6]^+$	259[79], 260[79], 261[79], 262[79], 263[79], 264[79], 265[79]	26	34
**2PG**	**2**	$[M]^+$ - $[M+2]^+$	155[79], 156[79], 157[79]	26	13
**PEP**	**3**	$[M]^+$ - $[M+3]^+$	167[79], 168[79], 169[79], 170[79]	18	22
**UDPG**	**6**	$[M]^+$ - $[M+6]^+$	565[323], 566[323], 567[323], 568[323], 569[323], 570[323], 571[323]	16	25
**ADPG**	**6**	$[M]^+$ - $[M+6]^+$	588[346], 589[346], 590[346], 591[346], 592[346], 593[346], 594[346]	16	25

Selected ion monitoring (SIM) with GC-MS			
**Metabolite**	**Mass range**	**Carbon atoms**	**Fragmentation**
**Serine**	*288-291*	2, 3	M-$C_7H_{15}O_2Si$
**Serine**	*302-305*	1, 2	M-$C_7H_{17}O_1Si$
**Serine**	*390-394*	1, 2, 3	M-C_4H_9
**Glycine**	*218-220*	2	M-C_5H_9O
**Glycine**	*246-249*	1, 2	M-C_4H_9
**Alanine**	*232-235*	2, 3	M-C_5H_9O
**Alanine**	*260-264*	1, 2, 3	M-C_4H_9
**Aspartate**	*316-320*	2, 3, 4	M-$C_7H_{15}O_2Si$
**Aspartate**	*390-394*	2, 3, 4	M-C_5H_9O
**Aspartate**	*418-423*	1, 2, 3, 4	M-C_4H_9
**Threinine**	*376-379*	2, 3, 4	M-C_5H_9O
**Threinine**	*404-408*	1, 2, 3, 4	M-C_4H_9
**Glutamate**	*330-334*	2, 3, 4, 5	M-$C_7H_{15}O_2Si$
**Glutamate**	*404-408*	2, 3, 4, 5	M-C_5H_9O
**Proline**	*258-262*	2, 3, 4, 5	M-C_5H_9O
**Asparagine**	*417-421*	1, 2, 3, 4	M-C_4H_9
**Asparagine**	*302-304*	1, 2	M-$C_8H_{18}NOSi$
**Glutamine**	*431-436*	1, 2, 3, 4, 5	M-C_4H_9
**Citrate**	*591-598*	1, 2, 3, 4, 5, 6	M-C_4H_9
**Succinate**	*289-294*	1, 2, 3, 4	M-C_4H_9
**Succinate**	*331-336*	1, 2, 3, 4	M-CH_3
**Fumarate**	*287-292*	1, 2, 3, 4	M-C_4H_9
**Malate**	*419-424*	1, 2, 3, 4	M-C_4H_9
**Malate**	*461-466*	1, 2, 3, 4	M-CH_3

Supplemental Table S3. Metabolite pool sizes. Metabolite pool sizes in Camelina single leaves were measured on the basis of leaf fresh weight (SD, n > 3). Both internal and external standards were used to calibrate the metabolite response curves and to correct for the loss of analyte, matrix contributions, and ion suppression.

**Compound**	**Levels** (nmol·gFW^{-1})
E4P	121.12 ± 21.14
RUBP	152.34 ± 9.43
3-PGA	415.40 ± 57.44
DHAP	81.58 ± 7.73
FBP	6.24 ± 0.87
G6P	149.01 ± 27.14
F6P	149.01 ± 27.14
G1P	36.29 ± 6.03
S7P	212.70 ± 35.45
R5P	25.81 ± 4.17
RU5P/XU5P	64.10 ± 9.71
UDPG	139.27 ± 18.99
ADPG	19.89 ± 8.53
Gln	3.64 ± 1.60
Glu	138.92 ± 46.80
Asp	50.16 ± 8.66
Asn	52.49 ± 14.65
Thr	294.24 ± 88.64
Ala	18.68 ± 4.43
Gly	58.46 ± 19.69
Ser	42.73 ± 9.86
Glycerate	206.67 ± 36.10
Glycolate	20.45 ± 57.55
Succinate	587.07 ± 154.48
Fumarate	3691.14 ± 478.48
Malate	6118.02 ± 1443.53
2PG	16.78 ± 2.20
PEP	9.56 ± 2.24
